# A review of machine learning methods to predict the solubility of overexpressed recombinant proteins in *Escherichia coli*

**DOI:** 10.1186/1471-2105-15-134

**Published:** 2014-05-08

**Authors:** Narjeskhatoon Habibi, Siti Z Mohd Hashim, Alireza Norouzi, Mohammed Razip Samian

**Affiliations:** 1 Faculty of Computing, Universiti Teknologi Malaysia, Johor, Malaysia; 2 School of Biological Sciences, Universiti Sains Malaysia, Penang, Malaysia; 3 Advanced Medical and Dental Institute, Universiti Sains Malaysia, Penang, Malaysia; 4 Centre for Chemical Biology, Universiti Sains Malaysia, Penang, Malaysia

**Keywords:** Protein solubility, Protein solubility prediction, In silico prediction, Recombinant protein expression, *Escherichia coli*, Machine learning, Bioinformatics, Computational biology

## Abstract

**Background:**

Over the last 20 years in biotechnology, the production of recombinant proteins has been a crucial bioprocess in both biopharmaceutical and research arena in terms of human health, scientific impact and economic volume. Although logical strategies of genetic engineering have been established, protein overexpression is still an art. In particular, heterologous expression is often hindered by low level of production and frequent fail due to opaque reasons. The problem is accentuated because there is no generic solution available to enhance heterologous overexpression. For a given protein, the extent of its solubility can indicate the quality of its function. Over 30% of synthesized proteins are not soluble. In certain experimental circumstances, including temperature, expression host, etc., protein solubility is a feature eventually defined by its sequence. Until now, numerous methods based on machine learning are proposed to predict the solubility of protein merely from its amino acid sequence. In spite of the 20 years of research on the matter, no comprehensive review is available on the published methods.

**Results:**

This paper presents an extensive review of the existing models to predict protein solubility in *Escherichia coli* recombinant protein overexpression system. The models are investigated and compared regarding the datasets used, features, feature selection methods, machine learning techniques and accuracy of prediction. A discussion on the models is provided at the end.

**Conclusions:**

This study aims to investigate extensively the machine learning based methods to predict recombinant protein solubility, so as to offer a general as well as a detailed understanding for researches in the field. Some of the models present acceptable prediction performances and convenient user interfaces. These models can be considered as valuable tools to predict recombinant protein overexpression results before performing real laboratory experiments, thus saving labour, time and cost.

## Introduction

In biotechnology, production of recombinant proteins is a crucial process in both biopharmaceutical industries and scientific research. So far, *Escherichia coli* (*E. coli*), a bacterium that requires simple conditions to grow is still the favoured host for cloning and overexpressing most proteins which are non-glycosylated and do not have many cysteine residues [1].

Even though logical strategies of genetic engineering are well established, such as strong promoters and codon optimization, protein overexpression is often, still an art. In particular, heterologous expression is often afflicted with low levels of production and insoluble recombinant proteins forming inclusion bodies (protein aggregations). Yet, there is no generic solution available to enhance heterologous overexpression. The use of fusion proteins can sometimes be more successful at the expense of decreased total yield as a result of the fusion partner production. Features that differentiate between proteins in the negative (non-expressed) and positive (expressed) classes might indicate sequence characteristics that could be modified in optimization, corresponding to what was attained with codon optimization, where sequences of gene are modified to become compatible with the translational apparatus [2]. As the host expresses the proteins, one cause of non-expression is the harmful interaction with the metabolism of the host [3].

For a given protein, the extent of its solubility can indicate the quality of its function. In general, over 30% of recombinant proteins are not soluble [4]. About 33 to 35 percent of all expressed non-membrane proteins are insoluble and about 25 to 57 percent of soluble proteins are prone to aggregate at higher concentrations [5]. For a determined experimental condition (i.e. temperature, expression host, etc.), the solubility of a protein is determined by its sequence [6].

The trial-and-error procedure of protein overexpression can be avoided by identifying the promising proteins to improve the experimental success rate [7]. There are two types of approach for predicting solubility of protein: sequence-based and structure-based. In the structure-based technique, the free energy difference between aggregation and solution phases is computed. This method demands experimentally obtained high resolutions 3D structures which are hard to acquire for aggregation-prone proteins. Hence, the sequence-based technique is a feasible and widely used method. Generally, the computational sequence-based prediction methods investigate the protein overexpression in *E. coli* at the normal growth temperature of 37°C [8].

The correlation of amino acid sequence and the tendency to form inclusion body was shown for the first time by Wilkinson and Harrison [9]. Later, numerous methods based on machine learning were proposed to predict the solubility of proteins merely from amino acid sequences [10].

Protein solubility prediction can be considered a binary classification task where a classifier should discriminate between soluble proteins (positive samples) and insoluble proteins (negative samples). There are several classification methods (learning algorithm) namely, decision tree (DT) (e.g. C4.5 [11]), k-nearest-neighbour (KNN) [12], neural network (NN) [13,14], support vector machine (SVM) [15], etc.

The learning algorithm (i.e. the classification method) is selected based on numerous factors, such as the number of existing examples in the dataset, the data type to be classified (e.g. symbolic or numeric), and the number of examples probable to be inaccurate or noisy. The level of preferred interpretability of the outcomes is another issue to be considered [16].

The majority of current methods use SVM to build the model of solubility [4]. Appropriate SVM models can often achieve better performance in classification of biological sequence compared to other machine learning-based approaches [1]. Each study employs a different set of features. Considering the model performance, different results are obtained, but 70% is a common accuracy in many studies [4].

To date, all of the prediction approaches examined a single system of protein expression, such as the *A. niger* or the *E. coli* system. The works of Hirose et al. [3,10] are exceptions that explored two different systems (*E. coli* and wheat germ).

Some of the suggested methods of prediction offer their work as widely accessible web servers [3,10,17-20].

In spite of more than two decades of research on the subject, there has been only one report, reviewing seven solubility prediction tools [21]. In their valuable review, the authors have compared seven existing prediction tools based-on the following factors: prediction accuracy, usability, utility, and prediction tool development and validation methodologies. Our aim is to evaluate and investigate all published methods to predict protein solubility, so as to offer a detailed as well as a general understanding for the researchers.

The organization of the paper is as follows. The major protein solubility prediction studies are reviewed in section 2, with emphasis on their datasets, features, feature selection methods, predictor models and performance results. Section 3 presents a discussion on the models details, the best models and the data challenge for solubility prediction task. Lastly, section 4 concludes the paper and proposes some future research directions.

## Review

The methods to predict solubility of protein based on the machine learning are summarized in Table 1 in a chronological order, descending from the most recent*.* Due to space limitation, the reported performance of the works and the features used in each work are shown in Table 2 and Table 3 respectively. More detailed descriptions of the works are presented in “Additional file 1”.

**Table 1:** A summary of key components of studies to predict protein solubility (in chronological order)

#	**Paper**	**Dataset(s)**	**Feature selection method(s)**	**Modeling technique(s)**	**Web server**
1	[7]	Bacterial protein sequences with ‘soluble’ and ‘insoluble’ in NCBI are selected randomly.	Wrapper: SVM	Support vector machine	-
		Size: 5692			
		Soluble: 2448			
		Insoluble: 3244			
2	[10]	HGPD	Filter: Student’s *t*-test	Two techniques:	ESPRESSO:
		*E. coli*		Support vector machine	http://mbs.cbrc.jp/ESPRESSO
		Size: 5100			
		Soluble: 1774			
		Insoluble: 3326			
		Wheat germ		Sequence pattern-based method	
		Size: 2939			
		Soluble: 1941			
		Insoluble: 998			
3	[5]	eSol	Two methods:	Random forest	ProS:
		Size: 1918	1. Filter: Student’s *t*-test		http://shark.abl.ku.edu/ProS/
		Soluble: 886	2. Wrapper: Random forest		
		Insoluble: 1032			
4	[8]	Four datasets:	-	Two methods:	SCM:
		Sd957		Support vector machine	http://iclab.life.nctu.edu.tw/SCM/
		Dataset Chan et al. [18] (Table 1, row 11)		Scoring card method (SCM)	
		Solpro			
		PROSO II			
5	[4]	eSol	-	Four techniques:	-
		Size: 1600		1. Support vector machine	
				2. Random forest	
				3. Conditional inference trees	
				4. Rule ensemble	
6	[6]	PROSO II	Wrapper	A two-layer model:	PROSOII:
				1. Layer 1: Parzen window + logistic regression	http://mips.helmholtz-muenchen.de/prosoII
				2. Layer 2: Logistic regression	
7	[22]	eSol	-	Decision tree	-
		Size: 1625			
		Soluble: 843			
		Insoluble: 782			
8	[23]	eSol	Wrapper: SVM	Support vector machine	-
		Size: 2159			
		Soluble: 1081			
		Insoluble: 1078			
9	[3]	HGPD	Filer: Student’s *t*-test	Random forest	-
		*E. coli*			
		Size: 7823			
		Soluble: 2796			
		Insoluble: 5027			
		Wheat germ			
		Size: 3955			
		Soluble: 2739			
		Insoluble: 1216			
10	[24]	SOLP	Seven methods:	Support vector machine	-
			1. Filter: Information gain		
			2. Filter: Gain ratio		
			3. Filter: Chi squared		
			4. Filter: Symmetrical uncertainty		
			5. Wrapper: ReliefF		
			6. Wrapper: SVM recursive feature elimination (SvmRfe)		
			7. Embedded: One attribute rule		
11	[16]	121genes from different species were expressed in 6 different vectors.	Feature selection package in LIBSVM: Filter (F-score) + Wrapper (SVM)	Support vector machine	-
		Size: 726			
		Soluble: 231			
		Insoluble: 236			
		Non-expressed: 259			
12	[20]	A database collected through literature search.	N/A	Logistic regression	http://www.biotech.ou.edu/
		Size: 212			
		Soluble: 52			
		Insoluble: 160			
13	[17]	Solpro	Wrapper	A two- layer model:	SOLpro:
				1. Layer 1: 20 Support vector machines	http://scratch.proteomics.ics.uci.edu
				2. Layer 2: One support vector machine	
14	[25]	eSol	Using histogram	Support vector machine	-
15	[19]	PROSO	Two methods:	A two-layer model:	PROSO:
			1. Wrapper	Layer 1: Support vector machine	http://mips.helmholtz-muenchen.de/proso/
			2. Filter: Symmetrical uncertainty	Layer 2: Naive Bayes	
16	[26]	Idicula‒Thomas 2006	N/A	Support vector machine	-
17	[27]	Idicula‒Thomas 2006	Filter: Unbalanced correlation score	Support vector machine	-
18	[28]	Idicula‒Thomas 2005	Filter: Mann–Whitney test	Discriminant analysis (A heuristic approach of computing solubility index (SI))	-
19	[29]	Genes of *C. elegans* with one expression vector and one *Escherichia coli* strain.	Filter: Linear correlation coefficient (LCC)	**-**	-
		Size: 4854			
		Soluble: 1536			
		Insoluble: 3318			
20	[30]	TargetDB	Wrapper: Random forest	Decision tree	-
		Size: 27,000			
21	[14]	SPINE	Wrapper	Decision tree	-
		Size: 562			
22	[31]	SPINE	Embedded: Decision tree	Decision tree	-
		Size: 356			
		Soluble: 213			
		Insoluble: 143			
23	[18]	Some genes *of E. coli* were expressed.	N/A	Regression	-
		Size: 100			
24	[9]	Some genes *of E. coli* were expressed.	N/A	Regression	-
		Size: 81			

**Table 2:** Reported prediction performances of the models (in chronological order)

**#**	**Paper**	**Accuracy**	**Area under curve**	**F-score**	**Gain**	**Mathew correlation coefficient**	**Precision**	**Recall**	**Sensitivity**	**Specificity**
1	[7]	0.88	-	-	-	0.76	-	-	-	-
2*	[10]	0.68	0.78	0.67	-	0.42	0.56	0.85	-	-
		0.75	0.75	0.82	-	0.42	0.79	0.86	-	-
3	[5]	0.84	0.91	-	-	0.67	-	-	0.82	0.85
4	[8]	0.84	-	-	-	-	-	-	-	-
5	[15]	0.90	-	-	-	-	-	-	0.80	0.80
6	[6]	0.75	-	-	1.69	0.39	0.65	0.76	0.73	
7	[22]	0.75	0.81	-	-	-	-	-	-	-
8	[23]	-	-	-	-	-	-	-	-	-
9*	[3]	0.71	-	-	-	-	0.47	0.67	-	-
		0.71	-	-	-	-	0.85	0.74	-	-
10	[24]	-	-	-	-	-	-	-	-	-
11	[1]	0.83	0.89	0.75	-	-	0.73	0.78	-	-
12	[20]	0.94	-	-	-	-	-	-	-	-
13	[17]	0.74	0.74	-	1.49	0.49	0.74	0.74	-	-
14	[25]	0.80	-	-	-	-	-	-	-	-
15	[19]	0.72	0.78	-	1.43	0.43	-	0.72	-	-
16	[26]	0.79	0.76	-	-	-	-	-	0.68	0. 85
17	[27]	0.74	-	-	-	-	-	-	0.57	0.81
18	[28]	0.72	-	-	-	-	-	-	-	-
19	[29]	**-**	**-**	**-**	-	-	-	-	-	-
20	[30]	0.76	-	-	-	-	-	-	-	-
21	[16]	0.63	-	-	-	-	-	-	-	-
22	[31]	0.65	-	-	-	-	-	-	-	-
23	[18]	-	-	-	-	-	-	-	-	-
24	[9]	0.88	-	-	-	-	-	-	-	-

a. *Results for *E. coli* and wheat germ are shown respectively.

**Table 3:** Features used to predict protein solubility

**#**	**Paper**	**Features**
1	[7]	1. 2-level triangle CGR
		2. Entropy of “2-level triangle CGR”
		3. Dipeptide composition based on a different mode of pseudo amino acid composition (PseAAC)
		4. Entropy of “dipeptide composition”
2	[10]	Same as row 9 (Reference [3])
3	[5]	1. Counts of aromatic amino acids
		2. Counts of buried amino acids
		3. Counts of hydrogen bonds
		4. Counts of leucine amino acid
		5. Counts of arginine amino acid
		6. Negative charge
		7. Surface composition of amino acids in intracellular proteins of Mesophiles (percent)
		8. Beta-strand indices for beta-proteins
		9. Flexibility parameter for two rigid neighbours
		10. Net charge
		11. Counts of nitrogen atoms
		12. Long range non-bonded energy per atom
		13. Isometric point (pI)
		14. Free energies of transfer of AcWl-X-LL peptides from bilayer interface to water
		15. Ratio of negative charge amino acids
		16. Ratio of net charge of protein
		17. Dependence of partition coefficient on ionic strength
4	[8]	Dipeptide composition (400 features)
5	[4]	1. Reduced features (39 features produced by pepstats):
		a. Molecular weight, number of residues, average residue weight, charge and isoelectric point
		b. For each type of amino acid: number, molar percent and DayhoffStat
		c. For each physicochemical class of amino acid: number, molar percent, molar extinction coefficient (A280) and extinction coefficient at 1 mg/ml (A280)
		2. Dimers (2400 features):
		a. Dimers amino acid frequencies which are computed considering gaps of 1–5 amino acid
		3. Complete set
		a. Reduced features + Dimers
6	[6]	1. Amino acid frequencies (18 features): R, N, D, C, Q, E, G, H, I, K, M, F, P, S, T, W, Y, V
		2. Dipeptide frequencies (13 features): AK, CV, EG, GN, GH, HE, IH, IW, MR, MQ, PR, TS, WD
7	[22]	1. Monomer, dimer and trimmers using 7 different alphabets (18 features)
		2. Sequence-computed features:
		a. Molecular weight
		b. Sequence length
		c. Isoelectric point
		d. GRAVY index
		3. Features used in Niwa et al. work [25]
		4. Combination of all the above features 1–3.
8	[23]	1. Coil
		2. Disorder
		3. Hydrophobicity
		4. Hydrophilicity
		5. β-turn
		6. α-helix
9	[3]	1. Nucleotide sequence information:
		a. 1-mer
		b. Frequencies of 64 codons (3-mer)
		c. GC-contents
		2. Amin acid sequence information:
		a. Polypeptide length
		b. Frequencies of 20 single amino acids (1-mer)
		c. Frequencies of 8 chemical property groups
		d. Frequencies of 5 physical property groups
		e. Repeat of amino acids
		f. Repeat of 8 chemical property groups
		g. Repeat of 5 physical property groups
		3. Amino acid structural information:
		a. Frequencies of single amino acids in surface area
		b. Frequencies of 8 chemical property groups in surface area
		c. Frequencies of 5 physical property groups in surface area
		d. Number of transmembrane regions
		e. Disordered regions:
		i. Number of occurrence
		ii. Length
		iii. Proportion
		f. Secondary structures:
		i. alpha-helix
		ii. Beta-sheet
		iii. Others
10	[24]	1497 features computed by Protein Feature Server (PROFEAT) [32]:
		1. Group 1:
		a. Amino acid composition
		b. Dipeptide composition
		2. Group 2: Autocorrelation 1
		a. Normalized Moreau-Broto autocorrelation
		3. Group 3: Autocorrelation 2
		a. Moran autocorrelation
		4. Group 4: Autocorrelation 3
		a. Geary autocorrelation
		5. Group 5:
		a. Composition
		b. Transition
		c. Distribution
		6. Group 6: Sequence order 1
		a. Sequence-order-coupling number
		b. Quasi-sequence-order descriptors
		7. Group 7: Sequence order 2
		a. Pseudo amino acid descriptors
11	[1]	1. Nucleotide information:
		a. 1-mer
		b. 2-mer
		c. 3-mer
		d. Sequence length
		e. GC content
		2. Amino Acid information:
		a. Features of Wilkinson and Harrison [9]
		b. Features of Idicula-Thomas et al. [27]
		c. Isoelectric point
		d. Peptide statistics
		3. Codon Adaptation Index
		4. PTMs
12	[20]	1. Molecular weight
		2. Cysteine fraction
		3. Hydrophobicity-related parameters:
		a. Fraction of total number of hydrophobic amino acids
		b. Fraction of largest number of contiguous hydrophobic/hydrophilic amino acids
		4. Aliphatic index
		5. Secondary structure-related properties:
		a. Proline fraction
		b. Alpha-helix propensity
		c. Beta-sheet Propensity
		d. Turn-forming residue fraction
		e. Alpha-helix propensity/b-sheet propensity
		6. Protein–solvent interaction related parameters:
		a. Hydrophilicity index
		b. pI
		c. Approximate charge average
		7. Fractions of: Alanine, Arginine, Asparagine, Aspartate, Glutamate, Glutamine, Glycine, Histidine, Isoleucine, Leucine, Lysine, Methionine, Phenylalanine, Serine, Threonine, Tyrosine, Tryptophan and Valine
13	[17]	1. Frequencies of amino acid monomers, dimers and trimmers using 7 different alphabets:
		a. Monomer frequencies
		i. [Natural-20:M]
		ii. [ClustEM-17:M]
		iii. [ClustEM-14:M]
		iv. [PhysChem-7:M]
		v. [BlosumSM-8:M]
		vi. [ConfSimi-7:M]
		vii. [Hydropho-5:M]
		b. Dimer frequencies
		i. [PhysChem-7:D]
		ii. [ClustEM-14:D]
		iii. [ClustEM-17:D]
		iv. [BlosumSM-8:D]
		v. [Natural-20:D]
		vi. [ConfSimi-7:D]
		c. Trimmer frequencies
		i. [ClustEM-17:T]
		ii. [Hydropho-5:T]
		iii. [ConfSimi-7:T]
		iv. [ClustEM-14:T]
		v. [Natural-20:T]
		2. Features computed directly:
		a. Sequence length
		b. Turn-forming residues fraction
		c. Absolute charge per residue
		d. Molecular weight
		e. GRAVY index
		f. Aliphatic index
		3. Predicted features using the SCRATCH suite of predictors:
		a. Beta residues fraction (Predicted by SSpro)
		b. Alpha residues fraction (Predicted by SSpro)
		c. Number of domains (Predicted by DOMpro)
		d. Exposed residues fraction (Predicted by ACCpro, using a 25% relative solvent accessibility cut-off)
14	[25]	1. Molecular weight
		2. Isometric point (pI)
		3. Ratios of each amino acid content
15	[19]	4. For mono-domain proteins:
		a. Word size 1:
		S, IL, M, F, DE, A, C, G, R
		b. Word size 2:
		R + R, R + C, R + E, R + T, N + Q, N + H, N + L, C + S, Q + A, Q + G, Q + I, E + A, E + G, E + K, E + P, E + V, G + P, H + M, L + Y, K + G, K + K, M + G, S + S, T + I, Y + C, Y + I
		c. Word size 3:
		ST + ST + ST, ST + ST + N, ST + DQE + AH, ST + C + ST, G + M + R, G + K + G, G + P + G,
		G + P + N, M + AH + AH, M + C + Y, DQE + G + R, DQE + R + DQE, DQE + M + ST,
		DQE + Y + N, DQE + AH + IV, K + R + IV, K + K + ST, P + DQE + DQE, P + DQE + C,
		IV + G + IV, L + IV + DQE, N + FW + DQE, N + C + P, AH + ST + ST, AH + K + L, C + FW + Y, C + K + C
		5. For multi-domain proteins:
		a. Word size 1:
		R, D, C, E, G, L, K, M, S, W
		b. Word size 2:
		A + Y, A + V, R + N, R + E, R + S, R + Y, N + A, D + M, C + T, Q + A, Q + E, E + D, E + G, E + T, G + I,
		G + F, G + S, H + C, H + M, H + P, L + G, L + S, K + D, K + G, K + L, K + F, P + L, T + L, T + Y, V + R
		c. Word size 3:
		ST + ST + ST, ST + P + DQE, ST + IV + K, R + DQE + FW, R + DQE + IV, R + IV + FW,
		FW + DQE + FW, M + ST + DQE, M + G + AH, M + FW + DQE, DQE + ST + ST,
		DQE + ST + G, DQE + G + K, DQE + IV + R, DQE + IV + L, P + G + ST, IV + ST + P,
		L + K + FW, AH + ST + IV, AH + G + IV, AH + AH + M
16	[26]	1. Aliphatic index
		2. Frequency of occurrence of residues Cysteine (Cys), Glutanic acid (Glu), Asparagine (Asn) and Tyrosine (Tyr)
		3. Reduced class of conformational similarity [CMQLEKRA]
		4. Reduced classes of hydrophobicity [CFILMVW] and [NQSTY]
		5. Reduced classes of BLOSUM50 substitution matrix [CILMV]
		6. The 18 dipeptide composition: [VC], [AE], [VE], [WF], [YF], [AG], [FG], [WG], [HH], [MI], [HK], [KN], [KP], [ER], [YS], [RV], [KY], [TY]
17	[27]	1. Physicochemical properties (6 features):
		a. Length of protein
		b. Hydropathy index (GRAVY)
		c. Aliphatic index
		d. Instability index
		e. Instability index of N-terminus
		f. Net charge
		2. Mono-peptide frequencies (20 features)
		3. Dipeptide frequencies (400 features)
		4. Reduced alphabet set (20 features)
18	[28]	1. Aliphatic index (AI)
		2. Instability index of the N terminus
		3. Frequency of occurrence of Asn, Thr, and Tyr
		4. Tri-peptide score
19	[29]	1. Signal peptide
		2. GRAVY
		3. Transmembrane helices
		4. Number of Cysteines
		5. Anchor peptide
		6. Prokaryotic membrane lipoprotein lipid attachment site
		7. PDB identity
20	[30]	1. General sequence composition
		2. Clusters of orthologous groups (COG) assignment
		3. Length of hydrophobic stretches
		4. Number of low-complexity regions
		5. Number of interaction partners
21	[16]	1. Single residue composition: I, T, Y
		2. Combined amino acid compositions: KR, DE, DENQ
		3. Predicted secondary structure composition: α and coil
		4. Presence of signal sequence
		5. Amino acid sequence length
		6. Number of amino acids in both short and long low complexity regions (over sequence length)
		7. Normalized low complexity value for both short and long regions (over sequence length)
		8. Minimum GES hydrophobicity score calculated over all amino acids in a 20 residue sequence window
22	[31]	1. Hydrophobe
		2. Cplx: a measure of a short complexity region based on the SEG program.
		3. Gln composition
		4. Asp + Glu composition
		5. Ile-composition
		6. Phe + Tyr + Trp composition
		7. Gly + Ala + Val + Leu + Ile composition
		8. His + Lys + Arg composition
		9. Trp composition
		10. Alpha-helical secondary structure composition
23	[18]	Same as row 24 (Reference [9])
24	[9]	1. Charge average approximation (Asp, Glu, Lys and Arg)
		2. Turn-forming residue fraction (Asn, Gly, Pro and Ser)
		3. Cysteine fractions
		4. Proline fractions
		5. Hydrophilicity
		6. Molecular weight (Total number of residues)

In the following tables, for an entry which does not have the corresponding column value, symbol “-” is used. For an entry which we could not find its value, but may exist, symbol “N/A” is used (N/A: Not applicable, not available or no answer).”

In order to comprehend the details of the works which are presented in Table 1, Table 2 and Table 3, datasets used, feature selection methods and performance measures are described in greater details in Table 4, Table 5 and Table 6 respectively.

**Table 4:** Databases/datasets used to predict protein solubility (in chronological order)

**#**	**Name**	**Reference**	**Size**			**Description**	**URL**
			**Total**	**Soluble**	**Insoluble**		
1	Sd957	[8]	957	285	672	It is made from 3 previous datasets: Idicula-Thomas et al. [28], Diaz et al. [20] and Chan et al. [1].	http://iclab.life.nctu.edu.tw/SCM/downloads.php
2	PROSO II	[6]	82,000	41,000	41,000	It is made from pepcDB and PDB and has been the largest dataset ever. It is balanced.	http://mips.helmholtz-muenchen.de/prosoII/img/Suppl_files.zip
3	HGPD	[33]	17,821 (As of June 9th, 2011)	N/A	N/A	Human full-length cDNA.	http://www.HGPD.jp
4	eSol	[25]	30,173	N/A	N/A	A database on the solubility of entire ensemble of *E. coli* proteins based on ASKA library.	http://www.tanpaku.org/tp-esol/index.php?lang=en
5	Solpro (SOLP)	[17]	17,408	8704	8704	It is collected from 4 different sources: PDB, SwissProt, TargetDB and dataset of “Idicula-Thomas, 2006”. The sequence redundancy is removed with 25% sequence similarity. It is balanced.	http://download.igb.uci.edu/SOLP.fa
6	PROSO	[19]	14,000	7000	7000	It is collected by merging 4 datasets: TargetDB, *PDB* and datasets of “Idicula-Thomas 2005” and “Idicula-Thomas 2006”.	-
7	pepcDB	[34]	N/A	N/A	N/A	It stored target and protocol information contributed by Protein Structure Initiative centres as well as targets imported from the TargetDB database. Now it has been replaced by TargetTrack.	http://pepcdb.rcsb.org
8	Idicula-Thomas 2006	[27]	192	62	139	It is collected from the literature.	-
9	Idicula-Thomas 2005	[28]	174	41	133	It is collected from the literature.	-
10	PDB	[35]	91,359 (As of 11 June 2013)	N/A	N/A	It is a repository of information about the 3D structures of large biological molecules, including proteins and nucleic acids.	http://www.rcsb.org/pdb/
11	SPINE	[16]	N/A	N/A	N/A	N/A	http://spine.nesg.org/user_login.cgi?url=http://spine.nesg.org/front_page.cgi?
12	TargetDB	[36]	295,041 (As of 29 March 2013)	N/A	N/A	It provided status information on target sequences and tracks their progress through the various stages of protein production and structure determination. Now it has been replaced by TargetTrack.	
							http://targetdb.rcsb.org
13	TargetTrack	-	316,424 (As of 14 June 2013)	N/A	N/A	It is a target registration database which provides information on the experimental progress and status of targets selected for structural determination by the Protein Structure Initiative and other worldwide high-throughput structural biology projects.	http://sbkb.org/tt

**Table 5:** **Description of feature selection methods used in machine learning**[37]

**Method**	**Description**	**Examples**
Filter	Filter methods evaluate the relatedness of features by looking at the inherent properties of the data. Usually a feature relevance score is computed, and the features with low scores are discarded.	Student’s *t*-test [N/A]
		Information gain [38]
		Gain ratio [38]
		Chi squared [N/A]
		Symmetrical uncertainty [39]
		Unbalanced correlation score [40]
		Mann–Whitney test [41]
		Linear correlation coefficient [N/A]
Wrapper	In wrapper methods various subsets of features are evaluated by training and testing a specific classification model, so a search algorithm is ‘wrapped’ around the classification model. This approach adapted to a specific classification algorithm.	Sequential forward selection [42]
		Sequential backward elimination [42]
		Beam search [43]
		ReliefF [44]
Embedded	Embedded methods, build the search for an optimal subset of features into the classifier construction, so they are specific to a given learning algorithm.	Random forest [45]
		SVM recursive feature elimination (SvmRfe) [46]
		One attribute rule [47]

**Table 6:** Performance measures used to evaluate protein solubility prediction (in alphabetical order)

**#**	**Name**	**Abbr.**	**Formula**	**Description**
1	Accuracy	ACC	(TP + TN)/(TP + TN + FP + FN)	The number of correctly classified instances divided by the total number of instances [6].
2	Area under ROC curve	AUC	-	It measures the discriminating ability of the model and it takes values between 0.5 for random drawing and 1.0 for perfect classifier [6].
3	Enrichment Factor	EF	[CS/(CS + WS)]/[S/(S + I)]	EF is especially suitable for the unbalanced datasets [27].
			CS: Number of correctly classified soluble proteins.	
			WS: Number of soluble proteins wrongly classified as insoluble.	
			S: total number of soluble proteins.	
			I: total number of insoluble proteins.	
4	False Negative	FN	-	The number of incorrectly predicted negatives [10].
5	False Positive	FP	-	The number of incorrectly predicted positives [10].
6	F-Score	FS	2 × Precision × Recall/(Precision + Recall)	The harmonic mean of recall and precision [10].
7	Gain	GAIN	Precision/proportion of the given class in the full data set.	It is an important performance measure that quantifies how much better the decision is in comparison with random drawing of instances [6].
8	Matthew’s Correlation Coefficient	MCC	(TP × TN - FP × FN)/((TP + FP)(TP + FN)(TN + FP)(TN + FN))	It indicates the correlation between the classifier assignments and the actual class in the two-class case. It is a good measure of classifier performance even when classes are unbalanced [6]. The MCC ranges between -1 and 1, and a large positive value indicates a better prediction [10].
9	Precision (Selectivity)	PRC	TP/(TP + FP) Or TN/(TN + FN)	The ratio of the number of correctly classified positive or negative instances to the number of all instances classified as positive or negative, for positive and negative class respectively [6].
10	ROC Curve	ROC	Plotting the “FP-rate” against the “TP- rate”, while the probability is increased from 0 to 1.0 with 0.01 increments.	The receiver-operator characteristic curve, showing the trade-off between the ratio of false positives and false negatives in testing a classifier [48]. A larger area value indicates a more robust prediction method [10].
11	Recall	REC	TP/(TP + FN)	The ratio of the number of correctly classified positive instances to the number of all instances from the positive class [6].
	(Sensitivity)			
	(True positive rate)			
	(TP- rate)			
12	Specificity	SPC	TN/(TN + FP)	The ratio of the number of correctly classified negative instances to the sum of all negative instances [6].
	(True Negative Rate)			
	(TN-rate)			
13	True Positive	TP	-	The number of correctly predicted positives [10].
14	True Negative	TN	-	The number of correctly predicted negatives [10].

a. “TP” = True Positive; “TN” = True Negative; “FP” = False Positive; “FN” = False Negative; “+” = Add, “-” = Subtract; “×” = Multiply; “/” = Division.

It should be mentioned that in some works several modeling techniques are examined and then one or more are selected as the final model(s). In the “Modeling Technique(s)” column of Table 1, only the final models are shown. It is same true for the “Feature Selection Method(s)” column. In addition, in most of the works, first an initial feature set is considered, and then using feature selection methods, a smaller sub-set is obtained and employed in the modeling. Table 3 presents the final sets used in the modelings.

With respect to the data used in each study, some of the authors created a dataset harvested from the literature, some employed public datasets, while others performed experiments to generate their own dataset.

## Discussion

This section investigates the works in more depth. In the following paragraph, the most used dataset, features, feature selection methods and learning techniques are presented. Afterwards, the best models based on the obtained accuracies are introduced. Then, the most convenient to use models are mentioned. Lastly, some data-related challenges are discussed.

In terms of data, eSol is the most widely used dataset in the field. Considering input features, the following features are the most common ones computed from the protein sequence: aliphatic index, amino acid sequence length, charge, amino acid compositions, instability, isoelectric point (pI), hydrophilicity, molecular weight, and predicted secondary structure. Filter methods (described in Table 5) are used more than the other feature selection techniques. Regarding the machine learning method, support vector machine is the most common technique to make prediction; random forest, decision tree and logistic regression are the next most common ones, respectively.

Based on the results, the method reported by Diaz et al. [20] obtained the best prediction accuracy (94%) on their generated dataset. Similar prediction accuracy was also reported by Samak et al. [4] with an accuracy of 90% on the eSol dataset, followed by the works of Xiaohui et al. [7], and Wilkinson and Harrison [9] with a prediction accuracies of 88% based on their generated datasets.

Comparing the different models in terms of convenience and ease of use, the ones with publicly accessible web servers can be considered the most convenient to use and evaluate. They are ProS [5], PROSOII [6], SCM [8], ESPRESSO [10], SOLpro [17], PROSO [19] and the model of Diaz et al. [20].

It seems that by using an appropriate dataset, as well as suitable machine learning techniques, reasonable prediction performance is attainable. In addition, feature selection methods can reveal, to some extent, influential factors on solubility and the sequence characteristics that could be modified in optimization.

Poor generalization ability is one of the limitations of sequence-based methods founded on a small dataset [35]. In general, extracting a reliable dataset, in terms of experimental conditions and expression system is challenging as the majority of databases that deliver the information on the solubility of proteins often do not provide comprehensive information about the experimental particulars of solubility assessment. Furthermore, researchers generally handle imbalanced (i.e. unequal number of soluble and insoluble samples) data when collecting protein solubility records. Consequently, numerous research teams used different methods to collect consistent datasets that divide proteins into insoluble and soluble categories [24,27].

It is worth mentioning that the datasets employed to build SOLpro [17] and PROSOII [6] were gathered by integrating different search results of TargetDB, Protein Data Bank (PDB), and Swiss-Prot database. Then, the proteins were categorized into insoluble and soluble samples according to the proteins’ annotations. Although these methods were best working when an appropriate experimental dataset did not exist, they might not be reliable completely. A soluble protein without appropriate annotation, for example, may be incorrectly categorized as an insoluble protein and vice versa. Furthermore, annotations from diverse databases may not be consistent. Clearly, it is desirable to have a large protein set with solubility determined based on experiment by a single reliable protocol [5].

## Conclusions

In this paper, the works to predict protein solubility prediction are reviewed in details. They are assessed and classified with regards to the datasets used, features used, feature selection methods, machine learning algorithms and performance results.

Since the early work of Wilkinson and Harrison [9], models later proposed became more complex in terms of dataset size, number and types of features employed, feature evaluation techniques and machine learning methods to make prediction. In general, the performances of the models have improved greatly as well.

Some of the models provide acceptable prediction performance (e.g. in terms of accuracy). Especially the ones with convenient user interfaces (e.g. web applications), can be considered valuable tools to anticipate recombinant protein overexpression results before performing real laboratory experiments. This capability will lead to significant reduction of labour, time and cost.

Generating larger and more accurate datasets, working on organisms other than *E. coli* and discovering other influential features, are some considerations for future directions in the protein solubility prediction field.

## Competing interests

The authors declare that they have no competing interests.

## Authors’ contributions

NH carried out the literature review studies and drafted the manuscript. SZMH and MRS conceived the idea of the study, and helped to draft the manuscript. AN helped to draft the manuscript. All authors read and approved the final manuscript.

## Authors’ information

NH received her M.Sc. in Artificial Intelligence from Isfahan University of Technology, Iran, in 2009 and B.Sc. in Software Engineering from the same university, in 2005. She is a faculty member of the Islamic Azad University (IAU) in Iran, since 2011. Presently she is pursuing Ph.D. in Computer Science at Universiti Teknologi Malaysia. Her research interests are bioinformatics, synthetic biology, artificial intelligence and machine learning.

SZMH is an Associate Professor at the Department of Software Engineering, Faculty of Computing, Universiti Teknologi Malaysia (UTM). She received her B.Sc. Degree in Computer Science from University of Harford, USA, M.Sc. in Computing from University of Bradford, UK and Ph.D. research in Soft Computing from University of Sheffield, UK. Her research interests are Soft Computing techniques and applications, System Development and Intelligent System. Currently she is the Deputy Dean of Academic, Faculty of Computing, UTM and a member of Soft Computing Research Group (SCRG), K-Economy, UTM.

AN received his M.Sc. in Computer Engineering from Islamic Azad University, Iran, in 2006 and B.Sc. in Computer Science from Yazd University, Iran, in 2003. He is a faculty member of the Islamic Azad University (IAU) in Iran, since 2007. Presently he is pursing Ph.D. in Computer Science at Universiti Teknologi Malaysia. His research interests focus on machine learning, pattern recognition and computer vision.

MRS received his Ph.D. from University of New South Wales, Australia, in Biotechnology. He is currently a faculty member (Professor) in the School of Biological Sciences, Universiti Sains Malaysia. The research in his laboratory focuses on molecular genetics and structural biology of proteins. He has published extensively in these areas.

## Supplementary Material

Additional file 1 In detailed descriptions of 24 studies to predict protein solubility during 1991–2014 (February). Click here for file
