# Supplementary material for: A review of machine learning methods to predict the solubility of overexpressed recombinant proteins in Escherichia coli
Source: BMC Bioinformatics. 2014 May 8;15:134. doi: 10.1186/1471-2105-15-134 (PMC4098780; doi:10.1186/1471-2105-15-134)
Supplement: Additional file 1 — In detailed descriptions of 24 studies to predict protein solubility during 1991–2014 (February). [file 1471-2105-15-134-S1.docx]

**A Review of Machine Learning Methods to Predict the Solubility of Overexpressed Recombinant Proteins in *Escherichia coli***

Detailed descriptions of 24 published works to predict protein solubility during 1991-2014 (February).

## (Harrison, 1991)

**Dataset**

- - 81 proteins

**Features**

- - Six amino acid-dependent features in declining order of their correlation with solubility:
    - Charge average approximation (Asp, Glu, Lys and Arg).
    - Turn-forming residue fraction (Asn, Gly, Pro and Ser).
    - Cysteine fractions.
    - Proline fractions.
    - Hydrophilicity.
    - Molecular weight (total number of residues).

**Predictor Model**

- - Regression model.

**Result**

- - Correlation with inclusion body formation is strong for the first two parameters but weak for the last four.

## (Davis, 1999)

- - This work is a revision of Wilkinson–Harrison solubility model.

**Dataset**

- - Around 100 proteins.

**Features**

- - The first two parameters of Wilkinson–Harrison model:
    - Charge average approximation (Asp, Glu, Lys and Arg).
    - Turn-forming residue fraction (Asn, Gly, Pro and Ser).

**Predictor Model**

- - A two-parameter version of the Wilkinson–Harrison statistical solubility model.

## (Christendat, 2000)

**Dataset**

- A frozen version of SPINE database.
- From *Methanobacterium thermoautotrophicum organism.*
- 143 insoluble and 213 soluble proteins.

**Features**

- 53 features in descending order:
  - Hydrophobe: It represents the average GES hydrophobicity of a sequence stretch, as discussed in the text - the higher this value the lower is the energy transfer.
  - Cplx: a measure of a short complexity region based on the SEG program.
  - Alpha-helical secondary structure composition.
  - Gln composition.
  - Asp+Glu composition.
  - Ile-composition.
  - Phe+Tyr+Trp composition.
  - Asp+Glu composition.
  - Gly+Ala+Val+Leu+Ile composition.
  - Hphobe.
  - His+Lys+Arg composition.
  - Trp composition.

**Predictor Model**

- Decision tree.
- The full tree had 35 final nodes.
- They also derived similar trees for expressibility and crystallizability, but the statistics for these were less reliable due to their smaller size and were not reported.

**Result**

- 65% overall accuracy in cross-validated tests.
- Proteins that fulfil the following conditions are insoluble:
  - More frequently contained hydrophobic stretches of 20 or more residues.
  - Had lower glutamine content (Q < 4%).
  - Fewer negatively charged residues (DE < 17%).
  - Higher percentage of aromatic amino acids (FYW > 7.5%).
- Proteins that fulfil the following conditions are soluble:
  - Do not have a hydrophobic stretch.
  - Have more than 27% of their residues in (hydrophilic) ‘low complexity’.

## (Bertone, 2001)

**Dataset**

- 562 proteins from the *Methanobacterium thermoautotrophicum* organism from SPINE database.
- To identify which proteins were used for this study, they constructed a ‘frozen’ version of the database at bioinfo.mbb.yale.edu/nesg/frozen.

**Features**

- 42 features in following table (plus the highlighted features in Table 1 in the paper).

| Feature | Description | Number |
| --- | --- | --- |
| *C*(*r*) | Single residue composition (occurrence over sequence length: *r* = A, C, D, E, F, G, H, [**I**], K, L, M, N, P, Q, R, S, [**T**], V, W, [**Y**] | 20 |
| *C*(*c*) | Combined amino acid compositions; c = [**KR**], NQ, [**DE**], ST, LM, [**FWY**], HKR, AVILM, [**DENQ**], GAVL, SCTM | 11 |
| *C*(*a*) | Predicted secondary structure composition: *a* = [α], β, [**coil**] | 3 |
| **[Signal**] | Presence of signal sequence | 1 |
| **[Length**] | Amino acid sequence length | 1 |
| **[CPLX(*x*)**] | Number of amino acids in low complexity regions; *x* = s (short), l (long) | 2 |
| **[CPLXn(*x*)**] | Normalized low complexity value (CPLX over sequence length); *x* = s (short), l (long) | 2 |
| [**Hphobe**] | Minimum GES hydrophobicity score calculated over all amino acids in a 20 residue sequence window | 1 |
| HP-AA | Number of amino acids within a hydrophobic stretch below a threshold of –1.0 kcal/mol | 1 |
| Total |  | 42 |

**Feature Selection**

- They used a genetic algorithm to search the space of possible feature combinations; the relevance of individual feature subsets was estimated with several machine learning methods, including decision trees and support vector.
- Selected features (highlighted in the above table):
  - Amino acids E, I, T and Y.
  - Combined compositions of basic (KR), acidic (DE) and aromatic (FYW) residues.
  - The acidic residues with their amides (DENQ).
  - The presence of signal sequences and hydrophobic regions.
  - Secondary structure features.
  - Low complexity elements.

**Predictor Model**

- Decision tree.
- 10-fold leave-one-out cross-validation is used.

**Result**

- Prediction success evaluated by cross-validation: 61–65%
- Solubility:
  - A high content of negative residues (DE > 18%).
  - Absence of hydrophobic patches.
- Insolubility:
  - Low content of aspartic acid glutamic acid, asparagines and glutamine (DENQ < 16%).

## (Goh, 2004)

**Dataset**

- 27267 protein sequences in TargetDB from multiple organisms.

**Feature**

- Refer to Table 1 in the paper:
  - General sequence composition.
  - Clusters of orthologous groups (COG) assignment.
  - Length of hydrophobic stretches.
  - Number of low-complexity regions.
  - Number of interaction partners.

**Feature Selection**

- Random forest.
- Features in in decreasing order of importance rank:
  - S: Serine percentage composition.
  - DE: The percentage composition of small negatively charged residues.
  - COG: conservation across organisms.
  - SCTM.
  - Length (amino acid residues).

**Predictor Model**

- Decision tree.
- Implemented using R package.

**Result**

- The average prediction success: 76%.
- They found that protein solubility is influenced by a number of primary structure features including (in decreasing order of importance) content of serine (S < 6.4%), fraction of negatively charged residues (DE < 10.8%), percentage of S, C, T or M amino acids, and length (< 516 amino acids).
- The most significant protein feature was serine percentage composition.

## (Luan, 2004)

**Dataset**

- Total: 10167 ORFs of C. elegans (with one expression vector and one Escherichia coli strain).
- Number of expressed proteins: 4854.
- Number of soluble proteins: 1536 (out of 4854).

**Features**

- They generated a database containing a variety of biochemical properties and predictions calculated from the sequences of each of the C. elegans ORFs.

**Feature Selection**

- 34 parameters were correlated to expression and solubility.
- Using the linear correlation coefficient (LCC).
- Top features:
  - Signal peptide.
  - GRAVY (Grand Average of Hydropathicity, an indicator for average hydrophobicity of a protein).
  - Transmembrane helices.
  - Number of cysteines.
  - Anchor peptide.
  - Prokaryotic membrane lipoprotein lipid attachment site.
  - PDB identity.

**Result**

- The most prominent protein feature was GRAVY (Grand Average of Hydropathicity, an indicator for average hydrophobicity of a protein). Solubility is inversely correlated to the hydrophobicity of the protein.
- Proteins homologous to those with known structures have higher chances of being soluble.
- Because signal peptide and transmembrane helices are hydrophobic in nature, the conclusion is that hydrophobicity is the most important indicator for heterologous expression and solubility of eukaryotic proteins in *E. coli*.

## (Idicula‐Thomas, 2005)

**Dataset**

- 4 datasets:
  - S (soluble): 25.
  - I (insoluble): 105.
  - T (test): soluble(15), insoluble(25).
  - PM: soluble(1), insoluble(3).
- The keywords soluble, inclusion bodies, *E. coli*, and overexpression was used to search PubMed to identify proteins that have been overexpressed in *E. coli* under normal growth conditions. Here, normal growth conditions imply 37°C, no solubility- enhancing or purification-facilitating fusion tags, or chaperone co-expression, absence of small molecule additives (L-arginine, sorbitol, glycylglycine, etc.), no prior heat-shock treatment, etc. Many of the proteins overexpressed in *E. coli* had an N terminus His tag, and these proteins were not used in creation of the data sets since His tags have been reported to influence the solubility of proteins on overexpression.

**Features**

- Datasets S, I and T were pooled together and analyzed for the significance of the following parameters:
  - Molecular weight.
  - Net charge.
  - Aliphatic index (AI).
  - Instability index of the protein IIP and of the N terminus IIN.
  - Frequency of occurrence of Asn (FN), Thr (FT), and Tyr (FY).
  - Dipeptide and tripeptide scores (SDP and STP).

**Feature Selection**

- 2 statistical tests were used:
  - Mann-Whitney test:
    - It is a nonparametric test and identifies the parameters that vary significantly between two data sets.
    - It was carried out using the software SPSS v.10.0 to test the statistical significance of the differences observed for some of the parameters between the two data sets S and I.
  - Discriminant analysis:
    - It works well on distributions that are normal, in identifying the independent variables/parameters that can help in classification of the data sets.
    - It was carried out using the software SPSS v.10.0 to identify features that significantly vary in the two data sets.
    - The analyses were done by stepwise method and forced-entry method, and the prediction accuracy was determined by leave-one-out cross-validation.
    - Certain parameters identified to be deviating significantly between the two data sets by the Mann-Whitney test may not be regarded as significant for classification of the data by discriminant analysis.
    - Since statistical classifiers will suffer from the bias introduced by these parameters, it is necessary to develop a heuristic algorithm which can handle these parameters in a manner such that overfitting is minimal.

**Predictor Model**

- Heuristic approach of computing solubility index (SI):
  - It is a formula based-on the following parameters which had the best classification accuracy (according to discriminant analysis):
    - Tripeptide score.
    - Aliphatic index.
    - Instability index of the N terminus.
    - Frequency of occurrence of the amino acids Asn, Thr, and Ty.
  - Jack-knife test and bootstrapping was used to evaluate the performance of SI on S dataset.

**Result**

- The model is compared with the Harrison’s model (Table 2 in the paper).
- Thermostability, in vivo half-life, Asn, Thr, and Tyr content, and tripeptide composition of a protein are correlated to the propensity of a protein to be soluble on overexpression in *E. coli*.

## (Idicula-Thomas, 2006)

**Dataset**

- 192 proteins: 62 soluble (S) and 130 insoluble (I), obtained similar to their previous work (Idicula‐Thomas, 2005).
- Training dataset: 128 proteins (87 insoluble and 41 soluble).
- Test dataset: 64 proteins (43 insoluble and 21 soluble).

**Features**

- - - (1) Six physicochemical properties:
      - L: Length of protein.
      - GRAVY: Hydropathic index.
      - AI: Aliphatic index.
      - II_P_: Instability index.
      - II_N_: Instability index of N-terminus.
      - NC: Net charge.
    - (2) Mono-peptide frequencies: 20.
    - (3) Dipeptide frequencies: 400.
    - (4) Tri-peptide frequencies: 8000.
    - (5) Reduced alphabet set: 20.

**Feature Selection**

- “Unbalanced correlation score” applied on the 446 features (1, 2, 3, 5).
- 20 selected features:

| **Rank** | **SVM model with 446 features** | **Correlation with**  **solubility** |
| --- | --- | --- |
| 1 | AI | P |
| 2 | Glu | P |
| 3 | His-His | P |
| 4 | Arg-Gly | P |
| 5 | Arg | P |
| 6 | Gly | N |
| 7 | IIP | P |
| 8 | NC | P |
| 9 | Asn-Thr | N |
| 10 | Arg-Ala | P |
| 11 | Cys | N |
| 12 | Met | N |
| 13 | Gln | P |
| 14 | Phe | N |
| 15 | Ile | P |
| 16 | Gly-Ala | P |
| 17 | IIN | P |
| 18 | Ser | N |
| 19 | Leu | P |
| 20 | Pro | N |

**Predictor Model**

- SVM, KNN and liner logistic regression were tried.
- 3 SVM models:
  - Frist model: The following procedure was employed:
    - (1) Get the protein sequence data.
    - (2) Assign labels.
    - (3) Convert all the sequences to their numerical equivalents.
    - (4) Scale the features to zero mean and SD 1.
    - (5) Partition the data as training and test sets.
    - (6) Run SVM classifier on training set.
    - (7) Run SVM classifier on the test set to assess the generalization.
  - Second model: steps 5–7 were done with only 20 features that were ranked at the top (for SVM model with 446 features) with unbalanced correlation score method. The classification accuracy for this was almost same (with 70 ± 1% classification).
  - Third model: The following procedure was employed:
    - (1) Steps (1)–(6) are same as earlier.
    - (2) Add random Gaussian Noise in a feature.
    - (3) Observe the change in SVM discriminant function value f(x) to check the sensitivity to solubility.
    - (4) Repeat this for all the features.
- To investigate the effect of sampling of proteins into the training and test datasets, 50 random splits of the datasets S and I into training and test datasets were created. No change was observed.
- Due to the fact that classes were imbalanced in the dataset, modelling were done by adding class-dependent weights to regularize the learning process in KNN and SVM. The results of both these classifiers were improved as compared with their non-weighted counterparts.

**Result**

| **Algorithm** | **# of features** | **Accuracy** | **Specificity** | **Sensitivity** | **Enrichment factor** |
| --- | --- | --- | --- | --- | --- |
| SVM | 446 (1,2,3,5) | **72** | **76** | **55** | **1.68** |
|  | 46 (1,2,5) | 66 | 48 | 48 | 1.48 |
|  | 8446 (1,2,3,4,5) | 67 | 67 | 50 | 1.52 |

- The results of the weighted classifiers:
  - Weighted_KNN: accuracy=72%, sensitivity=57%, specificity=57%, enrichment factor=1.78.
  - Weighted_SVM: accuracy=74%, sensitivity=57%, specificity=81%, enrichment factor= 1.78.
- The method is able to predict correctly the increase⁄decrease in solubility upon mutation.

## (Smialowski P. M.-G., 2007)

The model called PROTO.

**Dataset**

- Around 14000 instances (half soluble and half insoluble) from merging 3 datasets:
  - TargetDB
  - PDB
  - Dataset of: (Idicula‐Thomas, 2005) & (Idicula-Thomas, 2006).
- The relationship between amino acid sequence and solubility may be significantly different between single- and multidomain proteins. In order to take into account these differences in the nature of folding/misfolding the datasets were split into the subsets of long multiple domain and short monodomain proteins.
- Since sequence length distributions were somewhat different for insoluble and soluble, the composition of sequence datasets was adjusted to account for this effect.

**Features**

- 1-mer and 2-mer frequencies.
- 1-mer, 2-mer and 3-mer frequencies of compressed alphabets (classified amino acids).

| **Clustering schema name** | **Based on the scale/matrix** | **Clustering method** | **Number of clusters** | **Amino acid groups** |
| --- | --- | --- | --- | --- |
| Sol14 | Combination of 8 protein solubility matrices | Expectation-Maximization | 14 | (S,T), (G), (R), (F,W), (M), (D,Q,E), (K), (Y), (P), (I,V), (L), (N), (H,A), (C) |
| Sol17 | Combination of 8 protein solubility matrices | Expectation-Maximization | 17 | (S), (H), (T), (L,I), (W), (M), (F), (D,E), (A), (C), (K), (G), (P), (Y), (N,Q), (R), (A) |

**Feature Selection**

- Wrapper method was used with the Naive Bayes method as a classification procedure and the ‘Best first’ approach as a search algorithm. The detailed procedure can be found in (Smialowski P. e., 2006).
- Additionally feature ranking was performed by measuring symmetrical uncertainty of attributes with respect to a given class (Hall, 2003). While selecting features, the grouping schema which performed best for a given word size was utilized.

| **Dataset** | **Word size** | **Grouping** | **Primary features selected** |
| --- | --- | --- | --- |
| Mono domain | 1 | Sol17 | S,IL,M,F,DE,A,C,G,R |
| Multiple domain | 1 | None | R,D,C,E,G,L,K,M,S,W |
|  |  |  |  |
| Mono domain | 2 | None | R+R,R+C,R+E,R+T,N+Q,N+H,N+L,C+S,Q+A,Q+G,Q+I,E+A,E+G,E+K,E+P,E+V,G+P,H+M,L+Y,K+G,K+K,M+G,S+S,T+I,Y+C,Y+I |
| Multiple domain | 2 | None | A+Y,A+V,R+N,R+E,R+S,R+Y,N+A,D+M,C+T,Q+A,Q+E,E+D,E+G,E+T,G+I,G+F,G+S,H+C,H+M,H+P,L+G,L+S,K+D,K+G,K+L,K+F,P+L,T+L,T+Y,V+R |
|  |  |  |  |
| Mono domain | 3 | Sol17 | ST+ST+ST,ST+ST+N,ST+DQE+AH,ST+C+ST,G+M+R,G+K+G,G+P+G,G+P+N,M+AH+AH,M+C+Y,DQE+G+R,DQE+R+DQE,DQE+M+ST,DQE+Y+N,DQE+AH+IV,K+R+IV,K+K+ST,P+DQE+DQE,P+DQE+C,IV+G+IV,L+IV+DQE,N+FW+DQE,N+C+P,AH+ST+ST,AH+K+L,C+FW+Y,C+K+C |
| Multiple domain | 3 | Sol14 | ST+ST+ST,ST+P+DQE,ST+IV+K,R+DQE+FW,R+DQE+IV,R+IV+FW,FW+DQE+FW,M+ST+DQE,M+G+AH,M+FW+DQE,DQE+ST+ST,DQE+ST+G,DQE+G+K,DQE+IV+R,DQE+IV+L,P+G+ST,IV+ST+P,L+K+FW,AH+ST+IV,AH+G+IV,AH+AH+M |

**Predictor Model**

- A two-level structure with an SVM on the first level and a naive Bayes classifier on the second level.
- The output of the primary classifier for each protein was obtained by 10-fold cross-validation and served as input for a secondary Naive Bayes classifier. A 10-fold stratified cross-validation over input data were performed to obtain class assignment for each protein and to estimate the accuracy of the second level classifier.

**Performance Evaluation**

- Performance of the first level classifier is calculated separately as well.
- The model is compared with the following previous works (Table 1):
  - Harrison’s model.
  - (Idicula‐Thomas, 2005)**.**
  - (Idicula-Thomas, 2006)**.**
- To check whether any of the following features could result in reasonably good classification performance, Naive Bayes classifier was trained and evaluated with these global features (Table 1 in the paper):
  - Sequence length.
  - Isoelectricpoint (pI).
  - Grand average of hydropathicity index (GRAVY).
  - Aliphatic index (AI).
  - Fold index (FI).
  - The combination of AI, FI, GRAVY and pI.
- Experimental verification: They tested their method against experimental data on solubility measured for 31 different constructs of two proteins as well.

**Result**

- Measures:
  - Accuracy
    - Positive class=74.9%.
    - Negative class=68.5%.
    - Average=71.7.
    - The statistical relevance of the results for both classes was very high with P-value <2.2E-16.
  - Recall
    - TP-rate=0.749.
    - TN-rate=0.685.
    - Average=0.717.
  - Gain
    - Positive class=1.408.
    - Negative class=1.463.
    - Average=1.435.
  - MCC=0.434.
  - AUC=0.781.
- The content of R, D, E, G, S, C, M and L to be relevant for the solubility of single and multiple domain proteins.
- Five dipeptide frequencies were the most important: RE, EG, KG, QA, HM.

## (Kumar, 2007)

**Dataset**

- The dataset of (Idicula-Thomas, 2006) were employed.
- This dataset consist of 192 protein sequences, 62 of which are soluble and the remaining 130 sequences form inclusion body.
- The instances were randomly divided into training and test sets keeping the inclusion body forming and the soluble proteins approximately in ratio of 2:1.
- The training dataset: 128 sequences, 87 inclusion body-forming and 41 soluble proteins.
- The test dataset: 64 sequences, 43 inclusion body forming and 21 soluble proteins.

**Features**

- The 446 features extracted:
  - Physiochemical properties: 6
    - Aliphatic index.
    - Instability index of the entire protein.
    - Instability index of the N-terminus.
    - Net charge.
  - Single amino acid residues arranged in alphabetical order (A,C,D): 20
  - 20 reduced alphabets:
    - 7 reduced class of conformational similarity.
    - 8 reduced class of BLOSUM50 substitution matrix.
    - 5 reduced class of hydrophobicity.
  - Dipeptide compositions: 400.

**Feature Selection**

- 27 features were found critical for predicting the solubility:
  - Aliphatic index.
  - Frequency of occurrence of residues Cysteine (Cys), Glutanic acid (Glu), Asparagine (Asn) and Tyrosine (Tyr).
  - Reduced class [CMQLEKRA] was selected from the seven reduced classes of conformational similarity.
  - From the five reduced classes of hydrophobicity originally reported, only [CFILMVW] and [NQSTY] were selected.
  - From the eight reduced classes of BLOSUM50 substitution matrix the only reduced class selected was [CILMV].
  - The 18 dipeptide whose composition were found to significant: [VC], [AE], [VE], [WF], [YF], [AG], [FG], [WG], [HH], [MI], [HK], [KN], [KP], [ER], [YS], [RV], [KY], and [TY].

**Predictor Model**

- Granular Support vector machines (GSVM).
- In this work association rules were used for the purpose of granulation.
- Before applying SVM, all the features were scaled by making their mean zero and standard deviation one.
- As the data was imbalanced, weighted SVM was used.
- The SVM parameters C,γ and weights were tuned by grid search.

**Performance Evaluation**

- The algorithm performance was subsequently tested on unseen test dataset using the same test measure as used by (Idicula-Thomas, 2006).
- 50 random splits of the dataset were taken (with the same ratio of nearly 1:2 between the two classes of proteins), and their average performance was measured.
- For an imbalanced data, receiver operation characteristic (ROC) curve is generally used as test measure.

**Result**

| Number of features | Algorithm | ROC | Accuracy (%) | Specificity(%) | Sensitivity(%) |
| --- | --- | --- | --- | --- | --- |
| 446 | SVM | 0.5316 | 72 | 76 | 55 |
| 446 | GSVM | 0.7227 | 75.41 | 81.40 | 63.14 |
| 27 | GSVM | **0.7635** | **79.22** | **84.70** | **68** |

- These results showed that the GSVM is capable of capturing inherent data distribution more accurately as compared to a single SVM build over complete feature space.
- The increased ROC showed that the model is not biased towards majority class and is capable of predicting the minority class (soluble proteins) as well with equally good accuracy.

## (Niwa, 2009)

- It has been a reference for too many other works.

**Dataset**

- The ASKA library (Kitagawa M, 2005) consists of all predicted ORFs of the *E. coli* genome, including membrane proteins.
- 4132 ORFs were synthesized in the cell-free translation system.
- They successfully quantified 70% of the *E. coli* ORFs (3,173 proteins of 4,132).

**Features**

- Molecular weight.
- Isometric point (pI).
- Ratios of each amino acid content.

**Predictor Model**

- A histogram of the data of 3,173 translated proteins, showed a clear bimodal, rather than normal Gaussian, distribution.
- They have done an extensive analysis to find out the relation between some properties and protein solubility, including:
  - Physicochemical Properties.
  - Secondary structures: They could not detect a notable correlation between the predicted secondary structure content and the solubility.
  - Tertiary structure: some of the SCOP folds are strongly biased to the aggregation propensity
  - Function of the protein: For example the structural component group and the Factor group, showed a strong bias to the high-solubility group.
- SVM was built using 1,599 samples. It was trained with 1,000 randomly chosen samples. The prediction accuracy was calculated by the other 599 samples.
- Using the KSVM library in the kernlab package with R software.

**Result**

- Accuracy = 80%.
- The aggregation propensity is most correlated with the structural classification of proteins.
- A combination of 3-dimensional structure prediction with other physicochemical properties might improve the solubility prediction.

## (Magnan, 2009)

**Dataset**

- SOLP/Solpro database.
- 17408 of non-redundant proteins expressed in E.coli.
- It was collected from 4 different sources:
  - PDB.
  - SwissProt.
  - TargetDB database.
  - Dataset of (Idicula-Thomas, 2006).
- The sequence redundancy was removed with 25% sequence similarity.
- The SOLP database is balanced and it contains an equal number of soluble and insoluble proteins.

**Features**

- 23 groups of features:
  - 21 groups are frequencies of amino acid monomers, dimers and trimmers using seven different alphabets, including the natural 20 amino acid alphabet and six reduced alphabets (Table 2 in the paper):
    - Monomer frequencies:
      - [Natural-20:M]
      - [ClustEM-17:M]
      - [ClustEM-14:M]
      - [PhysChem-7:M]
      - [BlosumSM-8:M]
      - [ConfSimi-7:M]
      - [Hydropho-5:M]
    - Dimer frequencies:
      - [PhysChem-7:D]
      - [ClustEM-14:D]
      - [ClustEM-17:D]
      - [BlosumSM-8:D]
      - [Natural-20:D]
      - [ConfSimi-7:D]
      - [Hydropho-5:D]
    - Trimer frequencies:
      - [ClustEM-17:T]
      - [PhysChem-7:T]
      - [Hydropho-5:T]
      - [ConfSimi-7:T]
      - [ClustEM-14:T]
      - [BlosumSM-8:T]
      - [Natural-20:T]
  - 1 group computed features directly computed from the sequence (Ahujaet al., 2006; Idicula-Thomas and Balaji, 2005; Idicula-Thomas et al., 2006; Wilkinson and Harrison, 1991):
    - Sequence length.
    - Turn-forming residues fraction.
    - Absolute charge per residue.
    - Molecular weight.
    - GRAVY index.
    - Aliphatic index.
  - 1 group predicted features predicted using the SCRATCH suite of predictors:
    - Beta residues fraction, as predicted by SSpro.
    - Alpha residues fraction, as predicted by SSpro.
    - Number of domains, as predicted by DOMpro.
    - Exposed residues fraction, as predicted by ACCpro, using a 25% relative solvent accessibility cutoff.

**Feature Selection**

- Wrapper method (Kohavi, 1997) was used.
- 3 sets were removed:
  - [Hydropho-5:D].
  - [BlosumSM-8:T].
  - [PhysChem-7:T].

**Predictor Model**

- 3 models were evaluated: KNN (Weka), NN (Weka), SVM (LIBSVM) (Table 4 in the paper).
- Parameters of each algorithm were tuned to maximize the accuracy computed by 10-fold cross-validation.
- Each feature was normalized to [−1, +1].
- Final model: After experimentation and feature selection, 20 primary SVM predictors were retained, associated with 20 different feature sets. The 20 probability estimates produced by the primary predictors and the normalized sequence length made up the 21 final inputs to the second stage SVM combiner.
- The reported evaluation measures were the means of the corresponding 100 values obtained from 10 independently performed 10-fold cross-validation experiments.

**Result**

- Accuracy=74% with a threshold of 0.5.
- The SD of the accuracy was 0.044, the SDs of the other measures were also very small and thus, were not reported. The small SDs attest to the stability of the method.
- The authors found that the best single group of features was the content of the 20 amino acids.
- Measures:
  - ACC=74.15
  - MCC=0.487
  - Recall
    - Soluble=0.681
    - Insoluble=0.803
    - Average=0.742
  - Precision
    - Soluble=0.775
    - Insoluble=0.715
    - Average=0.745
  - Gain
    - Soluble=1.550
    - Insoluble=1.431
    - Average=1.490
  - AUC=0.742

## (Diaz, 2010)

- In this paper the biological reasons behind each selected feature and the other related issues have been described extensively.

**Dataset**

- Literature searches were done to find studies where the solubility or insolubility of a protein expressed in *E. coli* was discovered, regardless of the focus of the article. Only proteins expressed at 37 °C without fusion proteins or chaperones were considered, and membrane proteins were excluded.
- In determining the sequence of each protein expressed, signal sequences that were not part of the expressed protein were excluded due to their hydrophobic nature.
- The database contains 160 insoluble proteins and 52 soluble proteins. Of these 212 proteins, 52 were obtained from the dataset of (Idicula‐Thomas, 2005).

**Features**

- Molecular weight.
- Cysteine fraction.
- Hydrophobicity-related parameters:
  - Fraction of total number of hydrophobic amino acids.
  - Fraction of largest number of contiguous hydrophobic/hydrophilic amino acids.
- Aliphatic index
- Secondary structure-related properties:
  - Proline fraction.
  - a-Helix propensity.
  - b-Sheet Propensity.
  - Turn-forming residue fraction.
  - Alpha-helix propensity/b-sheet propensity.
- Protein–solvent interaction related parameters:
  - Hydrophilicity index.
  - pI.
  - Approximate charge average.
- Fractions of: Alanine, Arginine, Asparagine, Aspartate, Glutamate, Glutamine, Glycine, Histidine, Isoleucine, Leucine, Lysine, Methionine, Phenylalanine, Serine, Threonine, Tyrosine, Tryptophan, Valine.

**Predictor Model**

- 2 different models were evaluated with SPSS:
  - Binomial logistic regression.
  - Discriminant analysis models.
- Before analysing, data were normalized.

**Result**

- Classification accuracies for the logistic regression model (average accuracy of prediction):

| Model | Soluble | Insoluble | Overall |
| --- | --- | --- | --- |
| Stepwise forward without interactions | 9.6 | 97.5 | 75.9 |
| Stepwise forward with interactions | 86.5 | 96.3 | 93.9 |

- Classification accuracies for the discriminant analysis model (average accuracy of prediction):

| Model | Soluble | Insoluble | Overall |
| --- | --- | --- | --- |
| Stepwise forward without interactions | 61.5 | 59.4 | 59.9 |
| Stepwise forward with interactions | 57.7 | 75 | 70.8 |

## (Chan, 2010)

The production efficacy of different vectors varies for different target proteins. Trial-and-error is still the common practice to find out the efficacy of a vector for a given target protein.

Two limitations of the previous studies are:

- Most previous works only focused on demonstrating important factors related to solubility prediction and mixed the cases of target genes in inclusion fraction and non-expression to form a negative set. However, recent research has reported that recombinant proteins expressed as inclusion bodies still keep biological activity than previously appreciated (Gonzalez-Montalban N, 2007). Thus, it is still significant to distinguish inclusion bodies from the negative set in previous studies.
- They just considered the sequence of the protein to be expressed.

In this study, authors applied machine learning to train prediction models to predict whether a pairing of vector-protein will express or not express in *E. coli*. For expressed cases, the models further predict whether the expressed proteins would be soluble. Unlike many previous works, these models consider the sequence of the target protein as well as the sequence of the whole fusion vector as the features.

**Dataset**

- 726 scenarios:
  - 121 target genes from different species.
  - 6 fusion tags: CBP, GST, NusA, His, MBP, and Trx.
  - Each gene expressed in 6 vectors, resulting in 726 scenarios.
- The constructs transformed into *E. coli* under the same standard experimental conditions, as well as in parallel. Host strains of *E. coli* used in this study were JM109(DE3) and BL21-CodonPlus(DE3).
- Soluble: 231, insoluble: 236, non-expression: 259.

**Features**

- The major factors were correlated to mRNA expression and stability, codon usage in *E. coli*, solubility of whole fusion vectors, and Post-Translational Modifications (PTMs) on recombinant proteins.
- 617 features were extracted from an entire recombinant fusion protein. They were divided into two groups with respect to nucleotide or protein levels. The first 87 features were generated from nucleic acid sequences of entire recombinant fusion genes. The rest 530 features were retrieved from protein sequences.

**Feature Selection**

- Feature selection package in LIBSVM was used to measure the importance of the features. Removing those less important features from the feature set, resulted in a lower accuracy. Hence, all 617 features were kept to maintain the performance.

| **Feature Type** | **Description** | **#** |
| --- | --- | --- |
| Nucleotide | <= 3-mer | 84 |
| Nucleotide | nt Seq Length | 1 |
| Nucleotide | GC Content | 1 |
| Code Preference | Codon Adaptation Index | 1 |
| Amino Acid | Wilkinson and Harrison (1991) | 6 |
| Amino Acid | Idicula-Thomas et al. (2006) | 444 |
| Amino Acid | isoelectric point | 1 |
| Amino Acid | peptide statistics | 8 |
| PTMs | Plewczynski et al. (2005) | 71 |
| **Total**: 617 | | |

**Predictor Model**

- All features were normalized to zero mean and Standard Deviation to 1.
- 3 models were developed: flatSVM, nestSVM , and hierSVM.
- LIBSVM package was used to implement the models.
- SVM models used RBF kernel function.
- For each of the 3 classes, the same proportion was present in each random partition to divide instances into m parts.
- In training and validation, m-fold cross-validation (leave-one-out) was used.
- The procedure of training and testing was repeated for n times. Finally, the performance results of these n repeats were averaged and their corresponding SDs were measured.
- flatSVM
  - A classifier to predict 3 classes: no-expression, soluble, insoluble.
  - By using three 1vs1 classifiers, the prediction class of an instance vector was determined by a majority voting.
  - 652 instance vectors were used on training and validation by 10-fold CVs.
  - The other unseen 74 instance vectors in training and validation were applied to evaluate the performance of trained classifiers.
- nestSVM
  - Two binary classifiers were trained with **distinct sets of features** to predict whether a recombinant fusion gene could be expressed and whether an expressed recombinant fusion protein would be soluble in *E. coli*.
  - For the first classifier (expression), instance vectors labelled with soluble and insoluble were treated as one class.
  - For expression:
    - 87 Features derived from entire recombinant fusion nucleic acid sequences: 84 k-mer frequency features, length, GC-content, and CAI.
  - For solubility:
    - The other non-overlapping 530 features in protein level.
- hierSVM
  - Class labels were considered as attribute vectors instead of arbitrary numbers and involved the concept of hierarchical classification method [32].
  - Attribute vectors of labels were encoded as <1, 0, 0, 1>, <0, 1, 0, 1>, and <0, 0, 1, 0> to illustrate soluble, insoluble, and non-expression.

**Performance Evaluation**

- Comparing 3 SVM models:
  - F1 score (proper for multi-class classifier).
  - To investigate difference between pairs of the three methods, Student’s t-test and Yule’s Q-statistic were conducted to demonstrate the relationship of diversity.
- Comparing with the previous works:
  - F score, PRC and ROC curves.
  - The classifiers reduced to distinguish soluble from non-soluble cases (including insoluble and non-expression cases.)

**Results**

- Comparing 3 SVM classifiers (Table 4 in the paper):
  - The performance of AdaBoost was shown as the baseline performance.
  - flatSVM with high F1 measure, F score, and accuracy, outperformed other methods.
  - Using Student’s t-test, the pair wise relationship of three proposed methods was investigated with respect to accuracy. Only flatSVM and nestSVM resulted in statistical significance with a p-value less than 0.05.


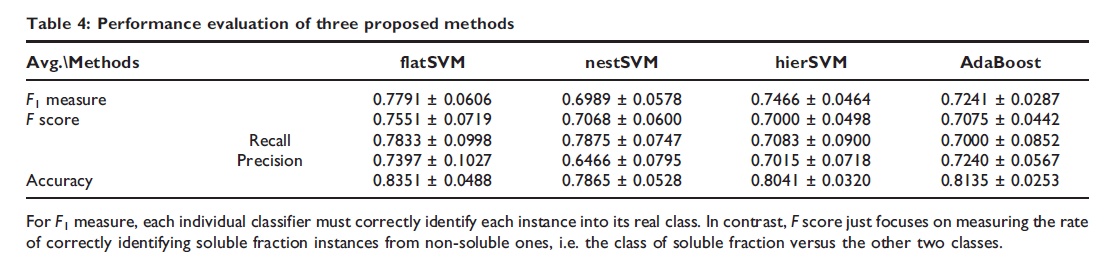


- Ensemble model:
  - Yule’s Q-statistic was calculated between pairs of proposed methods. The results (Table 6 in the paper) indicated that each method permit to train a classifier in a partial uncorrelation. This outcome showed the importance to combine different methods into an ensemble one.
- AUC= 0.8891.

## (Kocbek, 2010)

This study aimed to explain the relationship between the number of samples and stability of seven different feature selection techniques for protein datasets.

Several predictions have been done, based-on the protein sequence, including the solubility prediction. Number of sequence-derived features, used as input to the prediction methods, can be very high and they can contain redundant information. Therefore, different feature selection techniques are often applied to select the most important descriptors. Stability of these methods is very important and removing or adding learning instances should not influence the feature subset selection.

**Dataset**

- SOLP.

**Features**

- 1497 features.
- Protein Feature Server (PROFEAT) was used to obtain the features (which have been previously used in protein functional and structural prediction studies).

| **#** | **Feature Group** | **Feature** | **No. of Descriptor** | **No. of Descriptor Values** |
| --- | --- | --- | --- | --- |
| G1 | Amino acid, dipeptide composition | Amino acid composition | 1 | 20 |
|  |  | Dipeptide composition | 1 | 400 |
| G2 | Autocorrelation 1 | Normalized Moreau-Broto autocorrelation | 8 | 240 |
| G3 | Autocorrelation 2 | Moran autocorrelation | 8 | 240 |
| G4 | Autocorrelation 3 | Geary autocorrelation | 8 | 240 |
| G5 | Composition, transition and distribution | Composition | 7 | 21 |
|  |  | Transition | 7 | 21 |
|  |  | Distribution | 7 | 105 |
| G6 | Sequence order 1 | Sequence-order-coupling number | 2 | 60 |
|  |  | Quasi-sequence-order descriptors | 2 | 100 |
| G7 | Sequence order 2 | Pseudo amino acid descriptors | 1 | 50 (sequence length (SL) >= 30)  20 + SL - 1 (SL < 30) |

**Feature Selection**

- Seven feature selection methods in the WEKA were used in this study:
  - Information Gain (IG).
  - ReliefF (RF).
  - Support Vector Machines Recursive Feature Elimination (SvmRfe).
  - Gain Ratio (GR).
  - Chi Squared (CS).
  - One attribute rule (OR).
  - Symmetrical Uncertainty (SU).
- There were 2 different groups of methods: univariate and multivariate.
- Top ranked features were:
  - Composition-transition-distribution group.
  - Quasi-sequence-order descriptors group.

**Stability Evaluation**

- For each of 7 methods, several “feature set” with different size (25-1475), was calculated for 2 random data samples from the dataset. Then the “overlap score” between 2 data samples, was computed for each feature set (Figure 1 in the paper). Finally using SVM, the accuracy of classification was calculated for each method and each feature set. SVM classifiers had similar results with the previous works, but the feature selection methods differed in terms of stability and feature set’ size. The best feature selection method was the one with the highest “overlap score” and smallest “feature set” size.
- Univariate methods outperformed the multivariate ones in the stability context. The only exception was OR which showed multivariate behaviour.
- Performances of the methods in the univariate group were comparable and there were no significant differences in the overlap score between them. They reached good stability score with lower number of attributes compared to the multivariate methods.
- This indicated that researchers should use the univariate methods if they want stable and robust feature selection methods when selecting low number of protein descriptors **in protein solubility prediction problem**.
- They reached good stability scores at around 100 selected features while the multivariate methods needed more than 1000 features to reach the same stability score.

## (Hirose S. Y., 2011)

In this study, the overexpression and the solubility of human full-length cDNA in *E. coli* and a *wheat germ* cell-free expression system were assessed and the influences of sequence and structural features on protein expression/solubility in each system was evaluated and a minimal set of features associated with them was estimated.

**Dataset**

- For each expression system, there were 2 datasets: Single and multiple; Single comprised sequences for which the protein expression and solubility were experimentally assessed just one time.
- Two statistical models built using sequence information only to distinguish between overexpression and low expression; and between soluble and insoluble proteins.
- Size for *E. coli*:
  - Single: 2725 (+), 4909 (-).
  - Multiple: 71 (+), 118 (-).
- Size for *Wheat Germ*:
  - Single: 2653 (+),1166 (-).
  - Multiple: 86 (+), 50 (-).

**Features**

- There were 437 features in 2 groups:
  - Sequence information derived from nucleotide and amino acid sequence (396 features):
    - Nucleotide:
      - Occurrence frequencies of four single nucleotides
      - 64 codons.
      - GC contents.
    - Amino acid:
      - Occurrence frequencies of 20 single amino acids
      - Chemical property groups (8 groups).
      - Physical properties (5 groups).
      - Repeat: maximum number of consecutive same amino acids or property groups.
    - The values of these features were computed for the entire chains and both terminal regions, defined as 60 bases (20 amino acid residues), because modification of the terminal regions influences protein expression and solubility.
    - The use of a His-tag fusion raises the possibility that the features in the N-terminal region of the in vivo *E. coli* expression system and the C-terminal region in the *wheat germ* cell-free expression system may not be evaluated properly. They considered the His-tag to have the same influence on any sequences, since they conducted the protein expression experiments under the same conditions. Therefore, they evaluated them under this hypothesis.
  - Structural information derived with several predictions using amino acid sequence (40 features):
    - Secondary structures (predicted by PHD): the ratio of each element to the entire chain was computed.
    - Transmembrane regions (predicted by TMHMM): the number of occurrences in the entire chain was computed.
    - Disordered regions (predicted by POODLE-L): their number of occurrences, lengths and proportions in relation to the entire chain were computed.
    - Occurrence frequencies of single amino acids.
    - Same property groups on the protein surface (The accessible surface area was predicted using RVPnet).

**Feature Selection**

- For all features, the statistical difference between positive and negative data was determined using the Student’s t-test. A difference of P < 0.05 was considered significant.
- *E. coli*:
  - Feature vector size in *E. coli* for solubility: 45
  - Nucleotide information:
    - No GC content or single nucleotide was selected in the in vivo *E. coli*, but 18 out of 61 codons were chosen to have significant contribution to protein expression. Only 3 rare-frequency codons in the *E. coli*, among eight tested, passed the Student’s t-test. Although it has been suggested that the codon usage influences protein expression, little correlation between rare codons and protein expression was detected in this study (Figure 4 in the paper).
  - The amino acid ‘sequence information’ showed effects at the protein solubility stage (Figure 4 in the paper). The statistical analysis indicated that an abundance of charged residues in the C-terminal region leads increase of protein solubility.
- *Wheat Germ*:
  - A significant difference was found in the number of features associated with protein solubility in the two expression systems.
  - In the *wheat germ* cell-free expression system, the number of structural features that passed the Student’s t-test was smaller than that in the in vivo *E. coli* expression system, along with the number of sequence features.

**Predictor Model**

- Random forest.
- Data set_M was used for estimating the features associated with the protein expression and solubility; data set_S was used for assessing whether a set of selected features corresponds to the general characteristics on a genomic scale.
- Two models were built using set_M:
  - First evaluation method: 5-fold cross validation using only set_M.
  - Second evaluation method: using set_S.
- The classification abilities of these models were compared with each other as well as with the Wilkinson and Harrison model.

**Result**

- The accuracies (proportions of correct prediction) was 71.4% for protein solubility.
- The accuracy of the model for data set_SE was 6.1% higher than of Wilkinson’s model.
- Almost all of the proteins were expressed in the *wheat germ* cell-free expression system. 65% of the proteins were soluble. The *wheat germ* cell-free expression system exhibited higher performance in obtaining soluble proteins.
- The key features in the two expression systems were different. In *E. coli*, the charge is important, but it has little influence on the solubility in the *wheat germ* cell-free expression system. The differences between the features in the two expression systems might be related to the translation speed.

| **Expression system** | **Data set** | **Recall** | **Precision** | **Accuracy** |
| --- | --- | --- | --- | --- |
| *E. coli* | Data set_ME | 0.673 (0.296) | 0.468 (0.429) | 0.714 (0.587) |
|  | Data set_SE | 0.424 (0.295) | 0.551 (0.432) | 0.671 (0.610) |
| *Wheat Germ* | Data set_MW | 0.736 (0.302) | 0.853 (0.897) | 0.714 (0.537) |
|  | Data set_SW | 0.892 (0.294) | 0.718 (0.846) | 0.682 (0.469) |

## (Agostini, 2012)

- The model called CCSOL.

**Dataset**

- Dataset: (Niwa, 2009) .
- Protein identifiers were collected from the Ensembl Bacteria Database: <http://bacteria.ensembl.org/index.html>
- The dataset is split into three subsets:
  - Most soluble (1081 entries, “head set”).
  - Least soluble (1078 entries, “tail set”).
  - All the other proteins (884 entries).

**Features**

- 28 physicochemical properties collected through literature search:
  - Alpha-helix.
  - Beta-sheet.
  - Beta-turn.
  - TOP-IDB.
  - B-Value.
  - UnfoldOverFold.
  - DisProt.
  - Coil.
  - Hydrophobicity.
  - A.A. composition in SwissProt.
  - Buried residues.
  - Polarity.
  - Relative mutability.
  - Transmembrane tendency.
- Physicochemical profiles were generated by a window of seven amino acids sliding from the N- to the C-terminus of the protein sequence.
- They built a SVM to identify properties that allow the best discrimination between the “head” and “tail” sets.
- In the selection process, the SVM compares each protein of one dataset with all the proteins in the other dataset. Proteins scoring above a given threshold (80%) are discriminated and removed from the original data set for the next iterative round.
- After eight iterations, only 200 proteins remained un-discriminated, and 11 physicochemical characteristics (Conchillo-Sole, 2007) were collected.

**Feature Selection**

- In order to reduce the number of variables and identify those that give the strongest signal, they generated 2048 SVMs (all the combinations of 11 scales) and ranked them according to their performances upon cross-validation.
- Six properties were identified:
  - Coil.
  - Disorder.
  - Hrophobicity.
  - Hydrophilicity.
  - β-turn.
  - α-helix.

**Predictor Model**

- SVM.
- In order to reduce the number of variables and identify the strongest ones, 2048 SVMs are generated (all the combinations of 11 scales) and ranked them according to their performances upon cross-validation.
- The leave-one-out cross-validation process was repeated 10 times with each of the 10 subsamples used exactly once as the validation data.
- Six identified properties:
  - Coil.
  - Disorder.
  - Hydrophobicity.
  - Hydrophilicity.
  - β-turn.
  - α-helix.

**Result**

- They found that the most important features were disorder, coil, hydrophilicity, b–sheet and a–helix.

## (Stiglic, 2012)

This paper presented an extension to Weka (VTDT method) and a study on visual tuning of decision tree classifiers. Although they did not expect significant differences in classification performance, the results demonstrated a significant increase of accuracy in less complex visually tuned decision trees.

A lot of experts in different domains prefer to have the final decision tree printed out on a sheet of paper. The result of the VTDT method is a decision tree that can be printed out on a single page or displayed on a computer screen without the need for scrolling or zoom-in.

**Dataset**

- eSol containing 1,625 proteins, 782 insoluble and 843 soluble.

**Features**

- 21 feature (Table 2 in the paper) which have shown to be influential in protein solubility prediction in previous works:
  - 1–18: mono-, di- and tri-mers using 7 different alphabets
  - 19: 4 sequence-computed features, i.e., molecular weight, sequence length, isolectric point and GRAVY index.
  - 20: features used in (Niwa, 2009).
  - 21: combines all features from the previous datasets.

**Predictor Model**

- 2 decision tree models: VTDT & J48.

**Performance Evaluation**

- Measurements:
  - Basic size related measures like width and height of decision tree in pixels.
  - Number of leaves.
  - Number of nodes.
  - Classification accuracy (ACC) (calculated using 20 runs of 10-fold cross-validation).
  - Area under ROC curve (AUC) (calculated using 20 runs of 10-fold cross-validation).
- The Wilcoxon signed ranks test was used to assess statistical significance of difference in performance and complexity of the decision tree.

**Result**

- Based on 20 runs of 10-fold cross validation:
  - ACC=0.75.
  - AUC=0.81.

## (Smialowski P. D.–a., 2012)

- Compared with the previously published research, PROSOII model was trained on five times more data than used by any other method before (82299 proteins).

**Dataset**

- The dataset includes 82000 proteins (Table S2 of the paper).
- It is made from 2 databases:
  - pepcDB database.
  - PDB:
    - Regarding the fusion proteins, there is no obvious way to find out whether they were co-expressed or expressed as single proteins and then mixed. Therefore they decided to keep them in their analysis.
- A separate data set was built to model the real-life class distribution with a ratio of 1 to 5 between soluble and insoluble proteins, as observed in the pepcDB database (releases between May and December 2010).
- Several restrictive pre-processing steps done on the data.
- In the process of careful and restrictive data selection from the pepcDB and PDB databases, they built the currently **largest** available (more than 82000 proteins) input data set used for model building and evaluation.

**Features**

- Amino acid sequences representation:
  - Frequencies of mono-peptides.
  - Frequencies of dipeptides.
- Global sequence features:
  - Length.
  - pI.
  - GRAVY.
  - AI.
  - FI.
  - Length, pI, GRAVY, AI, FI.

**Feature Selection**

- The Wrapper method was used. The details are described in (Smialowski P. M.-G., 2007).
- A set of the best performing k-mers of length 1 and 2 was selected using the wrapper method.
  - There were 18 amino acid frequencies that were correlated with protein solubility: R, N, D, C, Q, E, G, H, I, K, M, F, P, S, T, W, Y, V.
  - 13 out of 400 dipeptide frequencies were selected as most important for model performance: AK, CV, EG, GN, GH, HE, IH, IW, MR, MQ, PR, TS and WD.
- The primary classifier, built on frequencies of dimers was the single best performing method.

**Predictor Model**

- The classification algorithm was organized as a two-layered structure: the output of a primary Parzen window model for sequence similarity and a logistic regression classifier of amino acid k-mer composition served as input for a second-level logistic regression classifier.
- Parzen Window approach: for each test protein, blastp scores (similarity) to the soluble and insoluble data sets (training data set, with 10-times cross-validation) were calculated. Then the probability of solubility was calculated using a formula.
- The threshold selector classifier (Frank E, 2004) (optimized for accuracy by an internal 10-fold cross-validation using only the training data) with a multinomial logistic regression model was used on both levels.
- They adjusted the threshold of the classifier to 0.6 using a separate data set, to account for non-equal distribution of soluble and insoluble instances in pepcDB. This threshold was selected to balance sensitivity and specificity.

**Performance Evaluation**

- Measurements:
  - AUROC: It was calculated using Weka.
  - Accuracy.
  - TP-rate.
  - TN-rate.
  - Specificity.
  - Precision.
  - Gain.
  - MCC.
  - Given the **unequal class distribution** in the holdout set **MCC** or **Gain** are much better suited to quantify classifier efficiency than accuracy.
- The SOLpro & CCSOL methods have been analysed and compared to this work too.

**Result**

- When tested on a separate real-lifelike class distribution holdout set, PROSO II attained the best results in comparison with other currently available methods:
  - Accuracy=0.75
  - MCC=0.39
  - Precision=0.655
  - Sensitivity=0.731
  - Specificity=0.759
  - Gain=1.692
- The performance of the method was only slightly dependent on the clustering level of the data (25%, 50%, 75% and 90%) (Table 2 in the paper).
- The “global sequence” features, and even their combination, did not perform well, so they believe that none of these simple features is significantly correlated with protein solubility.
- The result of feature selection combined with relatively low performance of the single amino acid based classification implies that protein solubility cannot be attributed to a single or a small group of amino acid frequencies.

**Limitations**

- PROSO II is only applicable to non-membrane proteins of size between 20 and 2004 residues.
- It is unable to take into account factors unrelated to protein sequence such as buffer composition, temperature or presence of nucleic acids.

## (Taghrid Samak, 2012)

In this work an analysis pipeline was proposed which is general and can be applied to any set of sequence features to predict any binary measure. The framework provides the biologist with a comprehensive comparison between different learning algorithms, and feature analysis.

Classification of a given numeric value as “soluble” or “insoluble” is very subjective, and uses domain knowledge. To arrive at a binary value, this work used a predefined threshold for solubility, which was decided by the biologist.

**Datasets**

- Dataset: eSol.
- Dataset size: over 1600.

**Features**

- Reduced features
  - 39 features.
  - Molecular weight, number of residues, average residue weight, charge, isoelectric point.
  - For each type of amino acid: number, molar percent, DayhoffStat.
  - For each physicochemical class of amino acid: number, molar percent, molar extinction coefficient (A280), and extinction coefficient at 1 mg/ml (A280).
  - This set was produced by pepstats.
- Dimers
  - 2400 features.
  - Dimers amino acid frequencies which were computed considering gaps of 1−5 amino acid.
- Complete set
  - Reduced features + dimers.

**Feature Selection**

- Unlike previous works, which mainly depended on individual correlations to evaluate features, their feature assessment was based on the generated models and the intersection of the best available accuracies.

**Predictor Model**

- The module supports four learning algorithms:
  - SVM: to compare the model with (Niwa, 2009).
  - Random Forests (RF): to avoid overfitting.
  - Conditional inference trees: to visual interpretation of features.
  - Rule ensemble method: to analysis a large family of proteins.
- Input dataset was split into train and set subsets. Different ratios were used to split the input dataset, where a random sample of the available data was selected as the training. For each sample size, multiple runs of the learning was performed, validated by the testing set, then the best model was stored in the model database. The information stored in the database also included the sample size, algorithm parameters, and the set of accuracy measures from the testing set.
- For each algorithm, different parameters can be passed to the module. For example, non-linear kernel for SVM, or the number of trees to grow in the forest.
- The main advantages of using Random Forests over SVM are the availability of ranking features based on their effect on solubility, and also the reduced overfitting effect.
- The pipeline provides information on feature importance, if available from the model (for example, Random Forests and Rule Ensembles).
- Pipeline was implemented using R package.

**Performance Evaluation**

- Measurements: accuracy, sensitivity and specificity.
- The size of the training dataset did not have a significant effect the performance. This was due to both the large total dataset size, and the diversity in the included protein families.
- They investigated whether there existed a set of proteins that were incorrectly mis-classified by all models using the same training parameters:
  - There existed a set of proteins that were incorrectly classified by all SVM models.
  - The results were different for Random Forests, where the mis-classified sets of proteins from each model were completely disjoint.
  - This observation might be an indication of the robustness of Random Forests in explaining the entire dataset.
  - It indicate that the mathematical models for SVM cannot explain certain biological information in those common proteins.

**Result**

- The 80% average accuracy has been previously reported using SVM by (Niwa, 2009). In this work the maximum accuracy of 90% is obtained using both SVM and Random Forests.

## (Hui-Ling Huang, 2012)

**Dataset**

- 4 datasets:
  - Sd957
    - 285 soluble proteins and 672 insoluble proteins
    - Collected mainly from three parts:
      1. (Idicula‐Thomas, 2005): A combination of the keywords inclusion bodies, soluble,E. coli, and overexpression was used to search PubMed for identifying proteins which have been overexpressed in E. coli under the normal growth condition. The proteins in this part have no fusion tags.
      2. (Diaz, 2010): The dataset of 212 proteins, including 52 soluble proteins and 160 inclusion bodies. The proteins in this part have no fusion tags.
      3. (named Sd726) (Chan, 2010): The dataset of 726 protein sequences which is the combination of six different fusion tags and 121 target proteins.
    - There are 980 proteins after integration of the three parts. After filtering by deleting duplicate proteins, 957 proteins remain in the final dataset.
    - The used dataset is available at <http://iclab.life.nctu.edu.tw/SCM/>
  - Sd726 (Chan, 2010)
  - SOLproDB
    - 17408 (8704 soluble and 8704 insoluble) proteins.
    - Although the study assumes that SOLproDB comes from the same experimental condition, the proteins from TargetDB possibly have ~20% of protein sequences which are expressed using different hosts.
    - After removing protein sequences which contain unknown amino acid residues, this dataset comprises 16902 (8212 soluble and 8690 insoluble) proteins.
  - SdPROSOII
    - The sequence identity of soluble and insoluble sets separately is further reduced at the sequence identity 25%.

**Features**

- Dipeptide composition.

**Predictor Model**

- Two methods:
  - SVM.
  - A novel scoring card method (SCM):

1. Creation of data sets for both training and independent test;
2. Establishment of an initial scoring matrix for propensity of dipeptides using a statistical approach;
3. Optimized the above matrix using Genetic algorithm;
4. Prediction of protein solubility using a formula.

- The dataset sd957 is used to for optimizing the solubility scoring matrix (SSM) and determining the suitable threshold value for classifying the query sequence as soluble or insoluble proteins.

**Performance Evaluation**

- 10-fold cross validation.
- SVM and SCM are compared using sd957.
- SCM is compared with the 3 prevoius works using their corresponding datasets Sd726, SOLproDB and SdPROSOII. For each specified data set, a scoring matrix of dipeptides is customized in the SCM method.

**Result**

- SVM:
  - Accuracy= 84.29.
- SCM:
  - Accuracy=84.29.
- Conclusion:
  - The classification method is very simple and the prediction result is easily interpretable. The SCM with SSMs performs well in predicting solubility, compared with existing complex methods using a large number of complementary features which correlate well with solubility.

## (Yaping Fang, 2013)

**Datasets**

- eSOL database.
- Only proteins with available sequences were retained.
- A protein with solubility <30% was considered as aggregation-prone and a protein with solubility >70% was considered as soluble.
- The set 30% identity made by CD-Hit.
- Size: 1918 proteins (886 soluble and 1032 aggregation-prone proteins).

**Features**

- 1438 features:

| Group | Protein features | Number of features |
| --- | --- | --- |
| I | Physicochemical properties obtained from AAindex | 544 |
|  | Density | 1 |
|  | Relative experimental aggregation propensities | 1 |
|  | Amyloid aggregation propensities | 1 |
|  | Solvent accessible area of exposed side chains | 1 |
|  | Property index | 12 |
| II | Number and composition of amino acids | 40 |
| III | Number and composition of dipeptides | 800 |
| IV | Sequence length (L) | 1 |
|  | Number and percentage of positive, negative and all charged residues, as well as the net charges | 8 |
|  | Number and percentage of small (T and D), tiny (G, A, S and P), aromatic (F, H, Y and W),  aliphatic, hydrophobic and polar residues | 12 |
|  | Number and percentage of residues which can form hydrogen bond in side chain | 2 |
|  | The average of the maximum solvent accessible surface area (ASA) of each amino acid | 1 |
|  | Predicted isoelectric point (pI) of protein, the average pI on all residues (pIa) | 2 |
|  | Instability index and instability class | 2 |
|  | Aliphatic index | 1 |
|  | Gravy hydropathy index | 1 |
|  | The overall length and percentage of all coils, rem465, and hotloop | 6 |
|  | Mean relative surface accessibility – RSA | 1 |
|  | Mean Z-fit score for RSA prediction | 1 |

**Feature Evaluation & Selection**

- Amino acid composition:
  - The statistical difference of amino acid composition between soluble and aggregation-prone proteins was estimated using the Student’s t-test.
  - Protein with more aromatic amino acid residues tended to be an aggregation-prone protein.
  - Soluble proteins tended to have more charged residues than aggregation-prone proteins.
- Performance of the feature sets
  - To estimate the importance and relevance of feature sets to the solubility, a series of models was built using different combinations of the four feature sets (Table 3 in the paper).
  - The model using all features achieved the best performance, suggesting that all features were relevant to protein solubility to some extent.
  - Different feature groups had different ability in classifying soluble and aggregation-prone proteins.
  - The amino acid composition features were most important and the dipeptide features were least important.
- To select the most informative and minimal subset features, the varSelRF package was used to iteratively eliminate 10% features for each iteration.
- 17 features were selected:

| **Feature** | **Annotation** | **Rank** |
| --- | --- | --- |
| c_aromatic | Counts of aromatic amino acids | 9 |
| c_bured | Counts of buried amino acids | 12 |
| c_Hbond | Counts of hydrogen bonds | 11 |
| c_L | Counts of leucine amino acid | 16 |
| c_R | Counts of arginine amino acid | 14 |
| FAUJ880112 | Negative charge | 6 |
| FUKS010102 | Surface composition of amino acids in intracellular proteins of mesophiles (percent) | 7 |
| GEIM800106 | Beta-strand indices for beta-proteins | 10 |
| KARP850103 | Flexibility parameter for two rigid neighbors | 15 |
| KLEP840101 | Net charge | 4 |
| num_n | Counts of nitrogen atoms | 13 |
| OOBM770103 | Long range non-bonded energy per atom | 17 |
| pI | Isometric point | 5 |
| WIMW960101 | Free energies of transfer of AcWl-X-LL peptides from bilayer interface to water | 1 |
| x_neg | Ratio of negative charge amino acids | 8 |
| x_netcharge | Ratio of net charge of protein | 3 |
| ZASB820101 | Dependence of partition coefficient on ionic strength | 2 |

- The prefix x represents the normalized absolute count values and c represents the absolute count values for each amino acid. The prefix num means the count of a specific atom. The other features are physicochemical properties of AAindex database.

**Predictor Model**

- Random forest model, consisting of 5000 decision trees.
- The number of variables randomly sampled in each tree was Radical M, where M is the number of total variables.
- The model was built on the 17-selected.

**Performance Evaluation**

- Performance with sequence identity:
  - To further evaluate the effectiveness of selected features, several models rebuilt at different sequence identity: 90%, 75%, 50% and 30%.
  - Results indicated that the selected features were effective and could be applied to build the models based on both strict and loose sequence identity.
  - A model using redundant-reduced dataset (identity<= 30%) achieved the same performance.
- The model compared with (Niwa, 2009) and (Stiglic, 2012).

**Result**

- Sensitivity = 0.82.
- Specificity = 0.85.
- Accuracy = 0.84.
- AUC = 0.91.
- MCC = 0.67.

## (Hirose S. a., 2013)

In this work (an enhancement of their previous work (Hirose S. Y., 2011)) an approach to estimate the probability of protein expression and solubility was proposed for two different protein expression systems: Escherichia coli and *wheat germ* cell-free, from only the sequence information.

It implemented two kinds of methods: a sequence/predicted structural property-based method, and a sequence pattern-based method that utilized the occurrence frequencies of sequence patterns.

**Dataset**

- Hirose (Hirose S. Y., 2011).
- The way of constructing the dataset was similar to their previous work.
- There were 2 datasets: single & multiple.
- The sequence identities in each dataset and between 2 dataset were 25%.
- Size for *E. coli*:
  - Single: 1705 (+), 3217 (-)
  - Multiple: 69 (+), 109 (-)
- Size for *Wheat Germ*:
  - Single: 1860 (+),949 (-)
  - Multiple: 81 (+), 49 (-)

**Sequence/predicted Structural Property-based Method**

- **Features**
  - - Similar to their previous work (Supplementary Information, Table 2, of the paper).
- **Feature Selection**
  - For each of the features, the statistically significant difference between the positive and negative datasets was computed by the Student’s t-test, using dataset_Multi. The features with p < 0.05 were considered to be associated with protein expression/solubility.
  - Selected features (Supporting Information, Figure 2):
    - - 50 features for solubility.
- **Predictor Model**
  - Three models were evaluated: SVM, RF and NN.
  - Selection of the machine learning model was done according to the F-score.
  - SVM was selected (Supporting Information, Table 3).
  - The parameters (cost and gamma) were optimized by 10-fold cross-validation.

**Sequence Pattern-based Method**

- **Features**
  - Defining the sequence patterns set:
    - It utilized the occurrence frequencies of highly frequent sequence patterns for prediction.
    - They estimated the set of sequence patterns associated with protein expression/solubility.
    - First, they defined the dataset of sequence patterns that were created by all combinations of ten amino acids, based on the physicochemical properties, with a length that exhibited the highest prediction performance.
    - Second, they searched for sequence patterns that only appeared in either the positive or negative data of dataset_Single, according to two scores: Score and p-value.
- **Predictor Model**
  - It was a discrimination function developed using the occurrence frequencies of sequence patterns:
    - SP_Score = (number of positive sequence patterns in a query) – [number of positive sequence patterns in the dataset / number of negative sequence patterns in the dataset] × (number of negative sequence patterns in a query)
    - If the value of the SP_score is larger than the threshold, then the query sequence is considered to be positive. The threshold was set to the value that showed the highest performance for the dataset_Single.

**Performance Evaluation**

- 5 criteria:
  - Recall.
  - Precision.
  - Accuracy.
  - F-score.
  - MCC.
  - AUC.
- The sequence/predicted structural property-based method was assessed by two approaches:
  - A 10-fold cross validation test using dataset_Multi.
  - An extension test in which the model trained by dataset_Multi was evaluated by dataset_Single.
- The sequence pattern-based method was assessed by using dataset_Multi. Since the set of sequence patterns was derived from dataset_Single, they are independent of dataset_Multi.

**Result**

- For protein solubility, the proposed method was compared with 3 publicly available servers: Wilkinson-Harrison model, PROSO, and SOLpro. The proposed method was superior regarding most of the criteria.
- For *E. coli*, the prediction performance of the property-based method was higher than that of the pattern-based method.
- Applying the proposed methods to genomic data revealed that proteins associated with translation or transcription have a strong tendency to be expressed as soluble proteins by the in vivo *E. coli* expression system. The sequence pattern-based method also had the potential to indicate a candidate region for modification, to increase protein solubility.


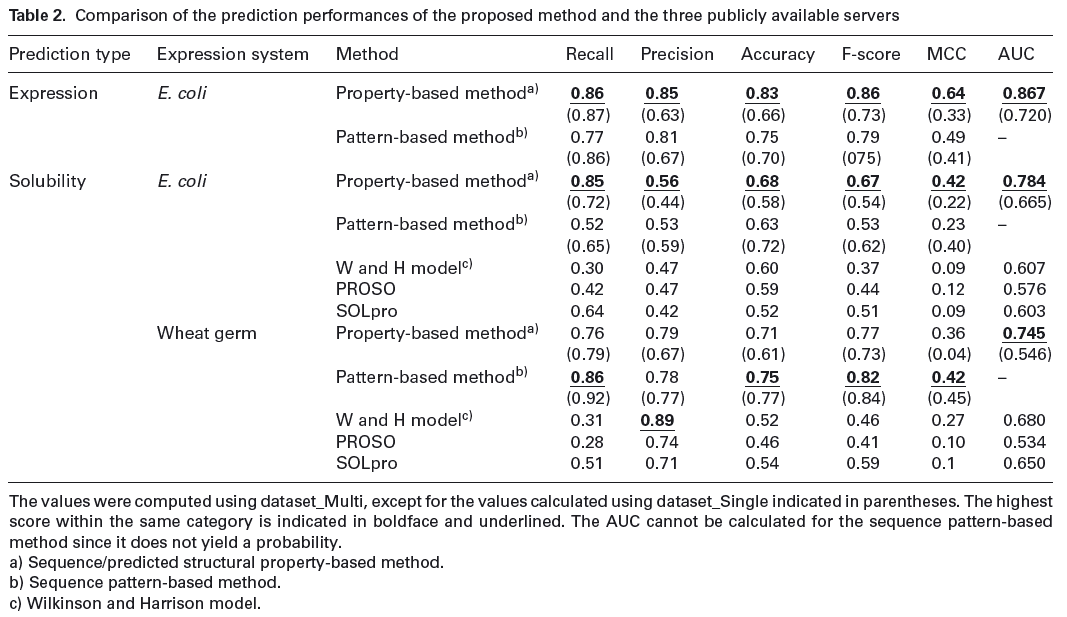


**Application to Model Organisms**

- - *E. coli* (strain K12) and Homo sapiens were selected as prokaryotic and eukaryotic representatives.
  - Their Protein data were downloaded from UniProt (release 2011_12). The *E. coli* and *H. sapiens* genomes contained 4430 and 20248 sequences.

## (Niu Xiaohui, 2014)

**Dataset**

- Dataset collection procedure:
  - They screened the related protein sequences with ‘soluble’ and ‘insoluble’ as in National Center for Biotechnology Information database (NCBI, http://www.ncbi.nlm.nih.gov/).
  - There were 69686 soluble proteins and 18034 insoluble proteins to hit.
  - In order to reduce the size of dataset, 5000 soluble bacteria protein sequences and 4500 insoluble bacteria nucleotide sequences were randomly picked out.
  - Finally, they removed the homologous sequences with the threshold (90% homologous similarity) by CD-HIT.
- Final dataset:
  - Size: 5692.
  - Soluble: 2448.
  - Insoluble: 3244.

**Features**

- Feature representation using:
  - Chaos Game Representation (CGR), and,
  - Entropy in information theory.
  - Based on a different mode of pseudo amino acid composition (PseAAC).
- There are 9 groups of candidate features:
  - AAC: amino acid composition.
  - Dipeptide.
  - Grid: 12-vertex polygon CGR.
  - Tri1: 1-level triangle CGR.
  - Tri2: 2-level triangle CGR.
  - Tri3: 3-level triangle CGR.
  - Quad1:1-level quadrangle CGR.
  - Quad2: 2-level quadrangle CGR.
  - Quad3: 3-level quadrangle CGR.
  - En_: Entropy derived from the corresponding feature vectors.

**Feature Evaluation**

- 3 different sets of features are evaluated by constructing several SVM classifiers:
  1. 9 groups of features, with and without the introduction of entropies (18).
  2. Pair-wise combination of the 9 groups of features (36).
  3. Pair-wise combination of the 9 groups of features and their corresponding entropies (36).

**Predictor Model**

- Predictor: SVM
  - With the most common kernel function: radial basis function (RBF).

**Performance Evaluation**

- Two approaches:
  - 10-fold cross-validation.
  - Re-substitution test.
- Measures: Sensitivity (SE), specificity (SP), accuracy (ACC) and Matthew’s Correlation Coefficient (MCC) value.

**Result**

- 10-fold cross-validation:
  - 2-level triangle polygon CGR + dipeptide composition together with their corresponding entropies as the mathematical feature.
    - ACC=**88.45%**, MCC=**0.7588**.
- Re-substituion test:
  - 3-level triangle polygon CGR, dipeptide composition and their entropies
    - ACC=92.38%, , MCC=0.8387.
- Conclusion:
  - Introduction of the entropy can significantly improve the performance of the classifiers.
  - Triangle CGR method surpass the two other CGR methods in classifier construction.
  - The optimal mathematical expression is dipeptide composition, triangle CGR and their entropies.
